# Small, Enigmatic Plasmids of the Nosocomial Pathogen, *Acinetobacter baumannii*: Good, Bad, Who Knows?

**DOI:** 10.3389/fmicb.2017.01547

**Published:** 2017-08-15

**Authors:** Soo Sum Lean, Chew Chieng Yeo

**Affiliations:** ^1^Saw Swee Hock School of Public Health, National University of Singapore Singapore, Singapore; ^2^Faculty of Medicine, Biomedical Research Centre, Universiti Sultan Zainal Abidin Kuala Terengganu, Malaysia

**Keywords:** *Acinetobacter baumannii*, small plasmids, antibiotic resistance genes, mobilizable plasmids, Rep-1 superfamily, Rep-3 superfamily, pRAY plasmids, toxin–antitoxin

## Abstract

*Acinetobacter baumannii* is a Gram-negative nosocomial pathogen that has become a serious healthcare concern within a span of two decades due to its ability to rapidly acquire resistance to all classes of antimicrobial compounds. One of the key features of the *A. baumannii* genome is an open pan genome with a plethora of plasmids, transposons, integrons, and genomic islands, all of which play important roles in the evolution and success of this clinical pathogen, particularly in the acquisition of multidrug resistance determinants. An interesting genetic feature seen in majority of *A. baumannii* genomes analyzed is the presence of small plasmids that usually ranged from 2 to 10 kb in size, some of which harbor antibiotic resistance genes and homologs of plasmid mobilization genes. These plasmids are often overlooked when compared to their larger, conjugative counterparts that harbor multiple antibiotic resistance genes and transposable elements. In this mini-review, we will examine our current knowledge of these small *A. baumannii* plasmids and look into their genetic diversity and phylogenetic relationships. Some of these plasmids, such as the Rep-3 superfamily group and the pRAY-type, which has no recognizable replicase genes, are quite widespread among diverse *A. baumannii* clinical isolates worldwide, hinting at their usefulness to the lifestyle of this pathogen. Other small plasmids especially those from the Rep-1 superfamily are truly enigmatic, encoding only hypothetical proteins of unknown function, leading to the question of whether these small plasmids are “good” or “bad” to their host *A. baumannii*.

## Introduction

*Acinetobacter baumannii* is a Gram-negative nosocomial pathogen that has become a serious healthcare concern especially in the last two decades due to its rapid ability to acquire antimicrobial resistance leading to the development of pandrug resistant (PDR) isolates that are resistant to all classes of antimicrobial compounds (Magiorakos et al., 2012; Göttig et al., 2014; Lean et al., 2014). Advances in genome sequencing and their increasing affordability have led to the availability of a plethora of *A. baumannii* genomes in the public databases (Peleg et al., 2012; Liu et al., 2013; Lean et al., 2015, 2016; Wallace et al., 2016). One of the key features of the *A. baumannii* genome is an open pan genome with a wide variety of mobile genetic elements, particularly integrons and transposons in genomic islands, some of which are known as resistance islands due to the presence of multiple antibiotic resistance genes (Fournier et al., 2006; Bonnin et al., 2012; Ramírez et al., 2013). Resistance genes are also plasmid-borne and in *A. baumannii*, plasmids range from as small as 2 kb to more than 100 kb in size (Gallagher et al., 2015; Hamidian et al., 2016a,b). The large plasmids of *A. baumannii* are often the focus of analyses due mainly to the presence of multiple antibiotic resistance genes and the self-transmissible nature of these plasmids (Hamidian et al., 2014a,b, 2016a; Hamidian and Hall, 2014) although small plasmids have been highlighted especially those that harbor antibiotic resistance genes (D’Andrea et al., 2009; Merino et al., 2010; Grosso et al., 2012; Hamidian et al., 2012, 2016b). Despite the importance of plasmids in the potential transmission of resistance and virulence genes in *A. baumannii*, there has been surprisingly very little experimental work done on the basic biology of these plasmids. We know next-to-nothing with regards to the basic replicons of these plasmids, their replication mechanisms and transmissibility. The rapidly increasing volume of *Acinetobacter* plasmid sequences in the databases from numerous whole genome sequencing projects has led to often conflicting and chaotic annotations, complicating their *in silico* analyses, a fact that was recently highlighted for all plasmid sequences in an excellent review paper by Thomas et al. (2017). So far, *A. baumannii* plasmids have been classified according to their replicase (Rep) proteins with Bertini et al. (2010) showing that there are 19 homology groups (GR1–GR19) and developing a plasmid-based replicon typing scheme based on their *rep* genes. In this mini-review, we shall examine our current knowledge of the small plasmids of *A. baumannii* (for this purpose, we shall define “small” as any plasmid that is around 10 kb and less) and present their genetic diversity and phylogenetic relationships. We will also discuss the importance of these small plasmids to their host *A. baumannii*.

## The Rep-3 Superfamily Plasmids

Majority of plasmids from *A. baumannii* encode replicase proteins belonging to the Rep-3 superfamily (identified by the pfam0151 conserved domain) with the larger plasmids usually harboring more than one replicon type (Bertini et al., 2010). In most of the Rep-3 superfamily replicons, the *rep* gene, which is usually annotated as *repB*, is preceded by three to six direct repeats (19–22 nucleotides in length and mainly located between 10 and 200 bp upstream of the *repB* start codon; majority are four direct repeats) that could be considered as the iterons for the RepB basic replicon (please see **Supplementary Table S1** and Data Sheet 1 for further details). In enterobacterial plasmids, these iterons serve as the origin of replication whereby the replication initiation protein binds and interacts with other host proteins (such as DnaA and the DnaBC helicase complex) required for replication initiation (Bertini et al., 2010; Konieczny et al., 2014). To the best of our knowledge, there has only been one experimental demonstration of the functionality of the *Acinetobacter* basic replicon. Dorsey et al. (2006) showed that the minimal replicon for the 9,540-bp plasmid pMAC from *A. baumannii* 19,606 was the *repB* gene [denoted as open reading frame-1 (ORF1)] and the four direct repeats that preceded the gene in experiments using the *Escherichia coli* cloning vector pCR-Blunt II-TOPO and *Acinetobacter calcoaceticus* BD413 as host.

**FIGURE 1:**
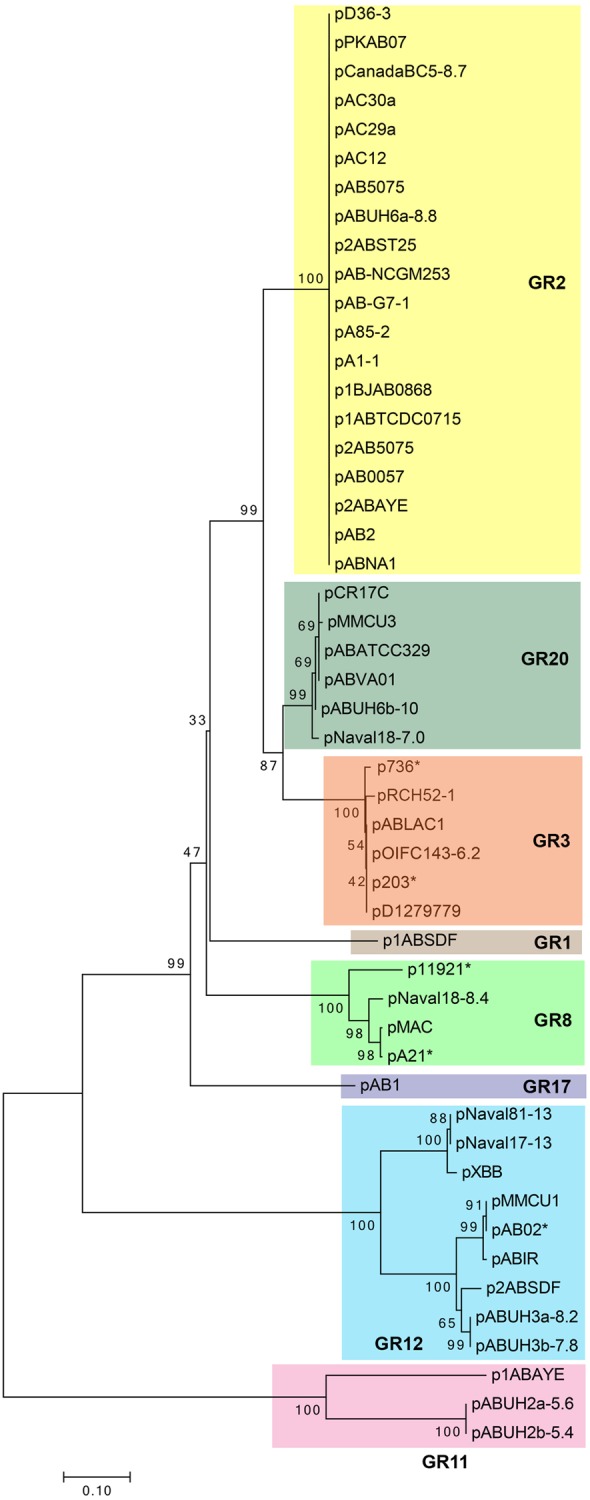
Phylogenetic tree of the small *Acinetobacter* plasmids of the Rep-3 superfamily based on the RepB replicase protein sequences as analyzed and drawn using MEGA7 (Kumar et al., 2016). Alignment of the RepB protein sequences was carried out using MUSCLE (Edgar, 2004) and the evolutionary history was inferred using the Neighbor-Joining method. The optimal tree with the sum of branch length = 3.32974406 is shown. The percentage of replicate trees in which the associated taxa clustered together in the bootstrap test (1000 replicates) is shown next to the branches. *(The tree is drawn to scale, with branch lengths in the same units as those of the evolutionary distances used to infer the phylogenetic tree. The evolutionary distances were computed using the Poisson correction method and are in the units of the number of amino acid substitutions per site. The analysis involved 50 RepB amino acid sequences with the GenBank accession numbers of the plasmids as listed in **Supplementary Table S1**. Each clade of the tree corresponded with the plasmid homology grouping (GR classification) as proposed by Bertini et al. (2010) and indicated by different colored boxes. Plasmid names marked with an asterisk (^∗^) indicate partial plasmid sequences that covered only the *oriV*–*repB* sequences and were included in the analysis to validate the plasmid groupings as they were used by Bertini et al. (2010) in their classification scheme.)*

Phylogenetic analysis using the RepB protein sequences of 50 of these Rep-3 superfamily plasmids (**Figure 1**) was largely in agreement with the plasmid homology groups proposed by Bertini et al. (2010). However, we are of the opinion that pABVA01 which was categorized under the GR2 group by Bertini et al. (2010) warrants a separate grouping along with similar plasmids such as pMMCU3 and pAbATCC329, which we designate GR20, as the phylogenetic tree clearly showed that this group of plasmids belonged to a separate clade (**Figure 1**).

Interestingly, in a majority of these small *A. baumannii* plasmids that belonged to the Rep-3 superfamily, the reading frame immediately downstream of the *repB* gene is highly conserved and is usually annotated as “*repA*” (**Supplementary Figure S1**). We could not find any homology to known replicase proteins for the translated “*repA*” gene and we are uncertain as to why this reading frame was designated *repA* in the absence of homology and/or experimental evidence. The translated protein contains a DNA-binding helix-turn-helix motif at its N-terminus and is usually annotated as a “conserved hypothetical protein” or a “DNA-binding protein” in the various database entries. The pMAC plasmid harbors this gene, which was designated ORF2, and which was shown by RT-PCR to be actively transcribed (Dorsey et al., 2006). Although for the pMAC plasmid, ORF2 was shown not to be part of the minimal replicon (Dorsey et al., 2006), its conservation in a vast majority of the small Rep-3 superfamily plasmids is suggestive of its importance. We have not found any evidence so far of the existence of any *Acinetobacter* plasmid that harbors only this “*repA*” reading frame without the *repB* gene. Nevertheless, a small number of *repB*-only plasmids do exist (such as p1ABAYE and the pABUH2a plasmids) and they form a distinct clade in the RepB phylogenetic tree (grouped under GR11; **Figure 1**) with their own unique iteron sequences (**Supplementary Table S1** and Data Sheet 1). Hence, in the absence of further experimental evidence, we could neither confirm nor completely rule out the involvement of this “*repA*” gene in the replication function of this group of plasmids. It is possible that some of these plasmids do require two replication genes, similar to IncQ plasmids such as RSF1010 which contained three replication genes with RepA functioning as the helicase, RepB as the primase, and RepC as the iteron-binding *oriV*-activator (Meyer, 2009).

Another key feature found in majority of the small Rep-3 superfamily plasmids is XerC/XerD recombination sites flanking various gene modules (**Supplementary Figure S1** and **Table S1**). Some of these gene modules include antibiotic resistance determinants such as *bla*_OXA-24_/*bla*_OXA-40_ (in pABVA01, pMMCU3, pAbATCC329, pABUH3a-8.2, and pABUH2a-5.6), *bla*_OXA-72_ (in p2ABST25 and pAB-NCGM253), and the *tet(39)* tetracycline-resistance gene (in pRCH52-1). XerC and XerD recombinases usually function to convert plasmid and chromosomal dimers to monomers during cell division with each recombinase catalyzing the exchange of a specific pair of strands between the recombining sites via a Holliday Junction, which is an essential reaction intermediate (Midonet and Barre, 2014). These recombinases are also involved in the integration of phage CTX-Φ in the *Vibrio cholerae* genome (Val et al., 2005) and transposition of certain conjugative transposons (Bui et al., 2006; Midonet and Barre, 2014). The DNA sequence of these small plasmids strongly infer the involvement of the XerC/XerD recombination system in the mobilization of discrete DNA modules, including antibiotic resistance genes, in *A. baumannii* although experimental proof of this has yet to be demonstrated.

Type II toxin–antitoxin (TA) systems are also found in most of the Rep-3 superfamily group of small plasmids. Type II toxin–antitoxin systems are known to mediate the stable maintenance of plasmids which harbor them through the post-segregational killing of any plasmid-free daughter cells that developed, making it difficult for the host cells to lose these plasmids (Hayes, 2003). Their presence may partly explain the widespread prevalence of this group of plasmids among *A. baumannii*. The AbkB/AbkA TA system (also known as SplT/SplA) has been shown to be a functional TA system with the AbkB (or SplT) toxin as an endoribonuclease and translational inhibitor, and AbkA (or SplA) as its cognate antitoxin (Jurenaite et al., 2013; Mosqueda et al., 2014). Other TA pairs found in these plasmids in place of AbkB/AbkA include RelE/Cro-CI (in pMAC, pABLAC1, and pD36-3), *phd–yoeB* (in p1ABAYE), *dinJ–yafQ* (in pABUH2 plasmids), and *rnlA–rnlB* (found flanked by XerC/XerD sites in pNaval18-7.0) (**Supplementary Table S1**). The functionality of these putative TA systems has yet to be experimentally verified.

Some of these Rep-3 superfamily small plasmids also harbor putative virulence factors in the form of a TonB-dependent receptor, septicolysin (Lean et al., 2016) and Sel1-repeat protein. TonB-dependent receptors are known to play a role in iron acquisition (Zimbler et al., 2013) whereas septicolysins are thiol-activated cytolysins with cytolytic activity toward eukaryotic cells and have been implicated in the pathogenesis of bacteria such as *Clostridium perfringens, Listeria monocytogenes*, and *Streptococcus pneumoniae* (Billington et al., 2000). Sel1-repeat proteins have diverse biological roles, often as adaptor proteins for the assembly of macromolecular complexes (Mittl and Schneider-Brachert, 2007). Bacterial Sel1-repeat proteins mediate interactions between the pathogen and its eukaryotic host cells and have been described in *Helicobacter pylori, Legionella pneumophila*, and *Pseudomonas aeruginosa* as important virulence factors, as reviewed in Mittl and Schneider-Brachert (2007). In *Neisseria meningitidis*, a Sel1-repeat protein, NMB0419, was shown to be involved in meningococcal interactions with epithelial cells (Li et al., 2003) and in a recent paper, it was intriguingly shown that the expression of NMB0419 led to transcriptional changes in genes involved in iron uptake, energy metabolism, and virulence functions in a manner counteracting the global regulator, Fur (Li et al., 2017). It would therefore be of interest to experimentally investigate if these genes encoded by some of the small plasmids of the Rep-3 superfamily truly function as virulence factors for *A. baumannii*, thereby contributing to the pathogenicity of the bacterium.

Some of these small Rep-3 superfamily plasmids also encode orthologs of the MobL or MobA mobilization proteins identified by the pfam03389 conserved domain found in the MobA/MobL protein family. Plasmids that encode genes for these proteins are mobilizable by other self-transmissible plasmids. Nevertheless, the only experimental evidence for the mobilization potential of these plasmids was for pMAC of *A. baumannii* 19606 with the experiment carried out using the cloned *mobA/mobL* gene in an *E. coli* DH5α host and an *E. coli* HB101 recipient (Dorsey et al., 2006). Until now, the mobilization potential of this group of plasmids from an *Acinetobacter* donor to an *Acinetobacter* recipient has yet to be shown.

## The Rep-1 Superfamily

There is a group of small cryptic plasmids from *A. baumannii* that usually comprise of a single *rep* gene and between two and five hypothetical genes. The *rep* gene of this group of plasmids encodes a replicase of the Rep-1 superfamily. Phylogenetic analysis of the Rep proteins from this group of plasmids showed that they could be divided into two subgroups: the p4ABAYE subgroup and the Rep63 subgroup (**Supplementary Figure S2**). The 2,726 bp p4ABAYE from *A. baumannii* AYE encodes a *rep* gene and four hypothetical ORFs (Fournier et al., 2006) and was categorized under the GR14 group of *Acinetobacter* plasmids (Bertini et al., 2010). The second subgroup contained two of the smallest reported *Acinetobacter* plasmids, the 1,967 bp p3AB5075 from *A. baumannii* AB5075 (Gallagher et al., 2015) and the 1,958 bp pM131-10 plasmid from *Acinetobacter* sp. M131 (accession no. JX101639). The small size of p3AB5075 has been validated by plasmid extraction and agarose gel electrophoresis (Gallagher et al., 2015), and the plasmid consisted of the *rep* gene and two other reading frames of unknown function. Although Gallagher et al. (2015) stated that the *rep* gene of p3AB5075 was of undefined plasmid replication group, our phylogenetic analysis indicated that it is grouped with an unpublished 2,343 bp *A. baumannii* plasmid pAB49 (accession no. L77992.1) (**Supplementary Figure S2**), which was previously categorized by Bertini et al. (2010) under the GR16 group. Furthermore, the *rep*-encoded protein of pAB49 had been previously shown to be homologous to the Rep63 replication initiation protein encoded by pBL63.1 of *Bacillus licheniformis* and orthologs in rolling-circle replicating (RCR) plasmids from various other bacterial species (Guglielmetti et al., 2005).

## Plasmid pRAY and its Derivatives

The 6,076 bp plasmid pRAY was first isolated from a South African clinical *Acinetobacter* strain designated SUN, which is of unknown clonal origin, through its carriage of the *aadB* gene which conferred resistance to the aminoglycosides gentamicin, kanamycin, and tobramycin (Segal and Elisha, 1999). The *aadB* gene is usually associated with class I integrons (Recchia and Hall, 1995), but in *Acinetobacter* sp. SUN and subsequently, in other *Acinetobacter* spp. isolated worldwide, *aadB* is found in pRAY and its closely related derivatives (Segal and Elisha, 1999; Adams et al., 2010; Nigro et al., 2011; Hamidian et al., 2012; Gifford et al., 2014; Ou et al., 2015; Kurakov et al., 2016). The *aadB* gene is likely acquired as its G+C content of 58% is higher than the G+C content of 37% for the rest of pRAY (Segal and Elisha, 1999) and the presence of an *attC* site immediately downstream of *aadB* is indicative of its gene cassette origin (Nigro et al., 2011).

A total of 10 ORFs, including *aadB*, was identified from the pRAY sequence, with two ORFs (designated ORF3 and ORF6) encoding proteins that were homologous to mobilization proteins (Segal and Elisha, 1999). A putative origin of transfer (*oriT*) was also identified upstream of ORF3 (Segal and Elisha, 1999), inferring the potential transmissibility of pRAY.

**FIGURE 2:**
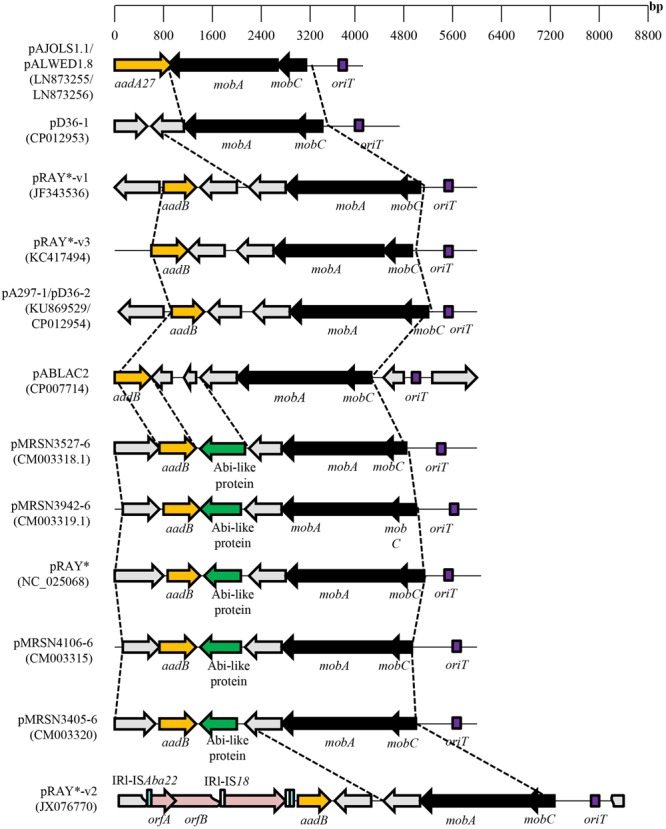
Comparative map of the pRAY plasmid and its derivatives from various *Acinetobacter* spp. The *mobA* and *mobC* genes are indicated as black arrows whereas the AT-rich putative *oriT* sequence is indicated as a purple box. Antimicrobial resistance genes (either *aadA27* for streptomycin/spectinomycin resistance or *aadB* for aminoglycoside resistance) are depicted as a dark yellow arrow while the Abi-like protein (identified by pfam07751) gene is indicated as a green arrow. IS element-encoded transposases are depicted in pink, the inverted repeats (IRs) for IS*Aba22* are shown as blue rectangles whereas the IRs for IS*18* are shown as white rectangles (for pRAY^∗^-v2). Hypothetical open reading frames are depicted as gray arrows. Accession numbers for the plasmids shown are in parentheses following their names.

Derivatives of pRAY have been characterized from Australian *A. baumannii* clinical strains with a plasmid designated pRAY^∗^ isolated from strain D36 and pRAY^∗^-v1 from strain C2 (Hamidian et al., 2012). The *mobA* gene from pRAY^∗^ is larger than ORF3 of pRAY but is still categorized within the ColE1 superfamily of MobA proteins (MOB_HEN_ family) with the putative *oriT* located upstream of *mobC* (Hamidian et al., 2012). Plasmid pRAY^∗^-v1 differed from pRAY^∗^ by 66 single nucleotide differences, 65 of which were within the *mobC*–*mobA* region leading only to amino acid substitutions of MobC and MobA but without any frameshifts. *A. baumannii* E7 harbored pRAY^∗^-v2 which was 2.5 kb larger than pRAY and sequence analysis indicated complete identity with pRAY^∗^ but with the insertion of two IS elements, an IS*18*-like element which is found within IS*Aba22* and located upstream of the *aadB* gene (Hamidian et al., 2012) (**Figure 2**). A single nucleotide variant of pRAY^∗^-v1, designated pRAY^∗^-v3, was isolated from a clinical strain of *A. nosocomialis* from Melbourne, Australia (Gifford et al., 2014).

Analysis of a 4,135 bp plasmid designated pALWED1.8 harbored in *A. lwoffii* isolated from the permafrost in Russia indicated conservation of the *oriT*–*mobC*–*mobA* region with pRAY and its derivatives (**Figure 2**) (Kurakov et al., 2016). The pALWED1.8 plasmid contained an *aadA27* gene downstream of *mobA* that conferred resistance to streptomycin/spectinomycin but without an *attC* site that was observed for *aadB* in pRAY and its variants (Nigro et al., 2011). Interestingly, the *oriT*–*mobC*–*mobA* backbone was identified from the genome sequence of various *Acinetobacter* species with various genes found in the accessory regions of these plasmids such as an alkyl sulfatase gene (involved in the degradation of surface-active substances such as sodium dodecylsulfate, or SDS) in the plasmid from *A. radioresistens* SK82 (Kurakov et al., 2016). Thus, members of this group of plasmids, including pRAY and pALWED1.8, might have originated from a common ancestor and independently acquired different genes into the accessory region of the plasmid. The mobilization of pALWED1.8 was demonstrated in conjugation experiments between *A. lwoffii* strain ED23-35 which contained pALWED1.8 and a large conjugative plasmid pKLH208 (Kholodii et al., 2004) and *A. baylyi* BD413rif as the recipient.

Intriguingly, until now, no potential replication initiation protein could be identified for pRAY and its derivatives based on sequence homology (Hamidian et al., 2012; Kurakov et al., 2016). Nevertheless, a potential origin of replication was identified for pRAY upstream of *aadB* where eight copies of an AT-rich repeat sequence, AAAAAATAT, were found (Segal and Elisha, 1999). The replication of these plasmids may mirror that of plasmids such as ColE1 which do not encode a *rep* gene since their replicon only consists of an *oriV* with the host RNA polymerase transcriptional machinery taking care of the melting of duplex DNA and synthesis of pre-primer RNA for replication initiation (Brantl, 2014; Thomas et al., 2017). Efforts to transform pRAY into *E. coli* were not successful, implying that pRAY and its derivatives might be specific for *Acinetobacter* (Segal and Elisha, 1999).

## Concluding Remarks

This mini-review has highlighted the small plasmids of *A. baumannii*, whether cryptic, resistance-related, or even mobilizable plasmids, and inferred the likely importance of these plasmids to their host. The potential of these small plasmids in transferring antibiotic resistance and possibly, even virulence genes, among *Acinetobacter* species should not be overlooked as their promiscuity could be comparable to that of larger plasmids and thus, would have a significant impact on the evolution of *A. baumannii*. The dearth of experimental studies with regards to these small *Acinetobacter* plasmids, given the importance of *A. baumannii* in the World Health Organization list of priority pathogens (World Health Organization, 2017), is indeed surprising and needs to be addressed. The PCR-based replicon typing (PBRT) scheme developed by Bertini et al. (2010) would probably need updating in view of an ever increasing amount of *A. baumannii* plasmid sequence data although their Rep-based classification scheme into different GR groupings appeared to be still valid with respect to the small plasmids. Nevertheless, plasmids of the pRAY-type would require another classification scheme due to the lack of a replicase protein. Other plasmid typing schemes such as plasmid multi-locus sequence typing (pMLST) and MOB classification based on plasmid mobility genes (Francia et al., 2004; Garcillán-Barcia et al., 2011) would be difficult to apply for these small *Acinetobacter* plasmids due to their lack of loci used in these typing schemes. There is clearly a need for us to accurately identify individual plasmids especially in this era of big data and whole genome sequencing (Orlek et al., 2017; Thomas et al., 2017), tracking the movement of plasmids and understanding their dynamic evolution, and small plasmids should not escape from our consideration simply because of their size.

## Author Contributions

SSL and CCY conceived, analyzed the data, wrote, edited, and approved this manuscript.

## Conflict of Interest Statement

The authors declare that the research was conducted in the absence of any commercial or financial relationships that could be construed as a potential conflict of interest.

## Supplementary Material

FIGURE S1 Comparative map of the small *Acinetobacter* plasmids of the Rep-3 superfamily. The *repB* replicase gene is indicated as a dark blue filled arrow, the putative *repA* gene is depicted in light blue. Hypothetical open reading frames are shown as unfilled arrows whereas black arrows are for the *mobA/mobL* mobilization genes. Red crosses indicate the XerC/XerD recombination sites. Filled blue twin-triangles depict the iterons that make up the putative origin of replication, *oriV*. Accession numbers and further details of the plasmids are as in **Supplementary Table S1** with detailed iteron sequences and locations on the respective plasmids in Supplementary Data Sheet 1.

FIGURE S2 Phylogenetic tree of the small *Acinetobacter* plasmids of the Rep-1 superfamily based on the Rep protein sequences, analyzed and drawn using MEGA7 (Kumar et al., 2016). Protein sequences were aligned using MUSCLE (Edgar, 2004), evolutionary history was inferred using the Neighbor-Joining method and the optimal tree (with the sum of branch length = 3.01508040) is shown. The percentage of replicate trees in which the associated taxa clustered together in the bootstrap test (1000 replicates) is shown next to the branches. The tree is drawn to scale, with branch lengths in the same units as those of the evolutionary distances used to infer the phylogenetic tree. The plasmids were grouped according to the GR classification scheme proposed by Bertini et al. (2010) and indicated here as GR14 and GR16 in different colored boxes. Accession numbers for the plasmids used in the analysis are as follows: p3AB5075 (NZ_CP008709.1), pBL63.1 (NC_006959.1), pM131-10 (NC_025169.1), pAB49 (L77992.1), pMRSN7339-2.3 (NZ_CM003313.1), p4ABAYE (NC_010403.1), pMRSN58-2.7 (NZ_CM003316.1), pA85-1 (NC_025107.1), and pTS236 (NC_016977.1). Note that pBL63.1 was isolated from *Bacillus lichineformis* and was included in the analysis based on the findings of Guglielmetti et al. (2005).

Table S1 Features of the Rep-3 superfamily group of *Acinetobacter* plasmids.

## References

[B1] Adams M. D. Chan E. R. Molyneaux N. D. Bonomo R. A. (2010). Genomewide analysis of divergence of antibiotic resistance determinants in closely related isolates of *Acinetobacter baumannii*. *Antimicrob. Agents Chemother.* 54 3569–3577. 10.1128/AAC.00057-10 20530228 PMC2934971

[B2] Bertini A. Poirel L. Mugnier P. D. Villa L. Nordmann P. Carattoli A. (2010). Characterization and PCR-based replicon typing of resistance plasmids in *Acinetobacter baumannii*. *Antimicrob. Agents Chemother.* 54 4168–4177. 10.1128/AAC.00542-10 20660691 PMC2944597

[B3] Billington S. J. Jost B. H. Songer J. G. (2000). Thiol-activated cytolysins: structure, function and role in pathogenesis. *FEMS Microbiol. Lett.* 182 197–205. 10.1111/j.1574-6968.2000.tb08895.x 10620666

[B4] Bonnin R. A. Poirel L. Nordmann P. (2012). AbaR-type transposon structures in *Acinetobacter baumannii*. *J. Antimicrob. Chemother.* 67 234–236. 10.1093/jac/dkr413 21965430

[B5] Brantl S. (2014). Plasmid replication control by antisense RNAs. *Microbiol. Spectr.* 2:PLAS-0001-2013 10.1128/microbiolspec.PLAS-0001-2013 26104196

[B6] Bui D. Ramiscal J. Trigueros S. Newmark J. S. Do A. Sherratt D. J. (2006). Differences in resolution of *mwr*-containing plasmid dimers mediated by the *Klebsiella pneumoniae* and *Escherichia coli* XerC recombinases: potential implications in dissemination of antibiotic resistance genes. *J. Bacteriol.* 188 2812–2820. 10.1128/JB.188.8.2812-2820.2006 16585742 PMC1446988

[B7] D’Andrea M. M. Giani T. D’Arezzo S. Capone A. Petrosillo N. Visca P. (2009). Characterization of pABVA01, a plasmid encoding the OXA-24 carbapenemase from Italian isolates of *Acinetobacter baumannii*. *Antimicrob. Agents Chemother.* 53 3528–3533. 10.1128/AAC.00178-09 19487447 PMC2715606

[B8] Dorsey C. W. Tomaras A. P. Actis L. A. (2006). Sequence and organization of pMAC, an *Acinetobacter baumannii* plasmid harboring genes involved in organic peroxide resistance. *Plasmid* 56 112–123. 10.1016/j.plasmid.2006.01.004 16530832

[B9] Edgar R. C. (2004). MUSCLE: a multiple sequence alignment method with reduced time and space complexity. *BMC Bioinformatics* 5:113 10.1186/1471-2105-5-113 PMC517706 15318951

[B10] Fournier P.-E. Vallenet D. Barbe V. Audic S. Ogata H. Poirel L. (2006). Comparative genomics of multidrug resistance in *Acinetobacter baumannii*. *PLoS Genet.* 2:e7 10.1371/journal.pgen.0020007 PMC1326220 16415984

[B11] Francia M. V. Varsaki A. Garcillán-Barcia M. P. Latorre A. Drainas C. De La Cruz F. (2004). A classification scheme for mobilization regions of bacterial plasmids. *FEMS Microbiol. Rev.* 28 79–100. 10.1016/j.femsre.2003.09.001 14975531

[B12] Gallagher L. A. Ramage E. Weiss E. J. Radey M. Hayden H. S. Held K. G. (2015). Resources for genetic and genomic analysis of emerging pathogen *Acinetobacter baumannii*. *J. Bacteriol.* 197 2027–2035. 10.1128/JB.00131-15 25845845 PMC4438207

[B13] Garcillán-Barcia M. P. Alvarado A. de la Cruz F. (2011). Identification of bacterial plasmids based on mobility and plasmid population biology. *FEMS Microbiol. Rev.* 35 936–956. 10.1111/j.1574-6976.2011.00291.x 21711366

[B14] Gifford B. Tucci J. McIlroy S. J. Petrovski S. (2014). Isolation and characterization of two plasmids in a clinical *Acinetobacter nosocomialis* strain. *BMC Res. Notes* 7:732 10.1186/1756-0500-7-732 PMC4210605 25326196

[B15] Göttig S. Gruber T. M. Higgins P. G. Wachsmuth M. Seifert H. Kempf V. A. J. (2014). Detection of pan drug-resistant *Acinetobacter baumannii* in Germany. *J. Antimicrob. Chemother.* 69 2578–2579. 10.1093/jac/dku170 24833751

[B16] Grosso F. Quinteira S. Poirel L. Novais Â. Peixe L. (2012). Role of common *bla*_OXA-24/OXA-40_-carrying platforms, and plasmids in the spread of OXA-24/OXA-40 among *Acinetobacter* species clinical isolates. *Antimicrob. Agents Chemother.* 56 3969–3972. 10.1128/AAC.06255-11 22526316 PMC3393421

[B17] Guglielmetti S. Mora D. Manachini P. L. Parini C. (2005). Genetic relationship among *Bacillus licheniformis* rolling-circle-replicating plasmids and complete nucleotide sequence of pBL63.1, an atypical replicon. *Plasmid* 54 93–103. 10.1016/j.plasmid.2005.01.002 16122558

[B18] Hamidian M. Ambrose S. J. Hall R. M. (2016a). A large conjugative *Acinetobacter baumannii* plasmid carrying the *sul2* sulphonamide and *strAB* streptomycin resistance genes. *Plasmid* 8 43–50. 10.1016/j.plasmid.2016.09.001 27601280

[B19] Hamidian M. Hall R. M. (2014). *pACICU2* is a conjugative plasmid of *Acinetobacter* carrying the aminoglycoside resistance transposon *TnaphA6*. *J. Antimicrob. Chemother.* 69 1146–1148. 10.1093/jac/dkt488 24335352

[B20] Hamidian M. Holt K. E. Pickard D. Dougan G. Hall R. M. (2014a). A GC1 *Acinetobacter baumannii* isolate carrying AbaR3 and the aminoglycoside resistance transposon Tn*aphA6* in a conjugative plasmid. *J. Antimicrob. Chemother.* 69 955–958. 10.1093/jac/dkt454 24235096 PMC3956371

[B21] Hamidian M. Holt K. E. Pickard D. Hall R. M. (2016b). A small *Acinetobacter* plasmid carrying the *tet39* tetracycline resistance determinant. *J. Antimicrob. Chemother.* 71 269–271. 10.1093/jac/dkv293 26416779 PMC4681370

[B22] Hamidian M. Kenyon J. J. Holt K. E. Pickard D. Hall R. M. (2014b). A conjugative plasmid carrying the carbapenem resistance gene *bla*_OXA-23_ in AbaR4 in an extensively resistant GC1 *Acinetobacter baumannii* isolate. *J. Antimicrob. Chemother.* 69 2625–2628. 10.1093/jac/dku188 24907141 PMC4164139

[B23] Hamidian M. Nigro S. J. Hall R. M. (2012). Variants of the gentamicin and tobramycin resistance plasmid pRAY are widely distributed in *Acinetobacter*. *J. Antimicrob. Chemother.* 67 2833–2836. 10.1093/jac/dks318 22888272

[B24] Hayes F. (2003). Toxins-antitoxins: plasmid maintenance, programmed cell death, and cell cycle arrest. *Science* 301 1496–1499. 10.1126/science.1088157 12970556

[B25] Jurenaite M. Markuckas A. Suziedeliene E. (2013). Identification and characterization of type II toxin-antitoxin systems in the opportunistic pathogen *Acinetobacter baumannii*. *J. Bacteriol.* 195 3165–3172. 10.1128/JB.00237-13 23667234 PMC3697630

[B26] Kholodii G. Mindlin S. Gorlenko Z. Petrova M. Hobman J. Nikiforov V. (2004). Translocation of transposition-deficient (TndPKLH2-like) transposons in the natural environment: mechanistic insights from the study of adjacent DNA sequences. *Microbiology* 150(Pt 4) 979–992. 10.1099/mic.0.26844-0 15073307

[B27] Konieczny I. Bury K. Wawrzycka A. Wegrzyn K. (2014). Iteron plasmids. *Microbiol. Spectr.* 2:PLAS-0026-2014 10.1128/microbiolspec.PLAS-0026-2014 26104462

[B28] Kumar S. Stecher G. Tamura K. (2016). MEGA7: molecular evolutionary genetics analysis version 7.0 for bigger datasets. *Mol. Biol. Evol.* 33 1870–1874. 10.1093/molbev/msw054 27004904 PMC8210823

[B29] Kurakov A. Mindlin S. Beletsky A. Shcherbatova N. Rakitin A. Ermakova A. (2016). The ancient small mobilizable plasmid pALWED1.8 harboring a new variant of the non-cassette streptomycin/spectinomycin resistance gene *aadA27*. *Plasmid* 84 36–43. 10.1016/j.plasmid.2016.02.005 26896789

[B30] Lean S. S. Suhaili Z. Ismail S. Rahman N. I. A. Othman N. Abdullah F. H. (2014). Prevalence and genetic characterization of carbapenem- and polymyxin-resistant *Acinetobacter baumannii* isolated from a tertiary hospital in Terengganu, Malaysia. *ISRN Microbiol.* 2014:953417 10.1155/2014/953417 PMC3977555 25006521

[B31] Lean S. S. Yeo C. C. Suhaili Z. Thong K.-L. (2015). Whole-genome analysis of an extensively drug-resistant clinical isolate of *Acinetobacter baumannii* AC12: insights into the mechanisms of resistance of an ST195 clone from Malaysia. *Int. J. Antimicrob. Agents* 45 178–182. 10.1016/j.ijantimicag.2014.10.015 25481460

[B32] Lean S. S. Yeo C. C. Suhaili Z. Thong K. L. (2016). Comparative genomics of two ST 195 carbapenem-resistant *Acinetobacter baumannii* with different susceptibility to polymyxin revealed underlying resistance mechanism. *Front. Microbiol.* 6:1445 10.3389/fmicb.2015.01445 PMC4700137 26779129

[B33] Li M. S. Farrant J. L. Langford P. R. Kroll J. S. (2003). Identification and characterization of genomic loci unique to the Brazilian purpuric fever clonal group of *H. influenzae* biogroup aegyptius: functionality explored using meningococcal homology. *Mol. Microbiol.* 47 1101–1111. 10.1046/j.1365-2958.2003.03359.x 12581362

[B34] Li M.-S. Langford P. R. Kroll J. S. (2017). Inactivation of NMB0419 encoding a Sel1-like repeat (SLR) protein in *Neisseria meningitidis* is associated with differential expression of genes belonging to the Fur regulon and reduced intra-epithelial replication. *Infect. Immun.* 85:e00574-16 10.1128/IAI.00574-16 PMC5400846 28264906

[B35] Liu C.-C. Tang C. Y. Kuo H.-Y. Lu C.-W. Chang K.-C. Liou M.-L. (2013). The origin of *Acinetobacter baumannii* TYTH-1: a comparative genomics study. *Int. J. Antimicrob. Agents* 41 318–324. 10.1016/j.ijantimicag.2012.12.010 23402702

[B36] Magiorakos A. Srinivasan A. Carey R. Carmeli Y. Falagas M. Giske C. (2012). Multidrug-resistant, extensively drug-resistant and pandrug-resistant bacteria: an international expert proposal for interim standard definitions for acquired resistance. *Clin. Microbiol. Infect.* 18 268–281. 10.1111/j.1469-0691.2011.03570.x 21793988

[B37] Merino M. Acosta J. Poza M. Sanz F. Beceiro A. Chaves F. (2010). OXA-24 carbapenemase gene flanked by XerC/XerD-like recombination sites in different plasmids from different *Acinetobacter* species isolated during a nosocomial outbreak. *Antimicrob. Agents Chemother.* 54 2724–2727. 10.1128/AAC.01674-09 20385865 PMC2876395

[B38] Meyer R. (2009). Replication and conjugative mobilization of broad host-range IncQ plasmids. *Plasmid* 62 57–70. 10.1016/j.plasmid.2009.05.001 19465049 PMC2752045

[B39] Midonet C. Barre F.-X. (2014). Xer site-specific recombination: promoting vertical and horizontal transmission of genetic information. *Microbiol. Spectr.* 2:MDNA3-0056-2014 10.1128/microbiolspec.MDNA3-0056-2014 26104463

[B40] Mittl P. R. E. Schneider-Brachert W. (2007). Sel1-like repeat proteins in signal transduction. *Cell. Signal.* 19 20–31. 10.1016/j.cellsig.2006.05.034 16870393

[B41] Mosqueda N. Gato E. Roca I. López M. de Alegría C. R. Fernández-Cuenca F. (2014). Characterization of plasmids carrying the *bla*_OXA-24/40_ carbapenemase gene and the genes encoding the AbkA/AbkB proteins of a toxin/antitoxin system. *J. Antimicrob. Chemother.* 69 2629–2633. 10.1093/jac/dku179 24879663

[B42] Nigro S. J. Post V. Hall R. M. (2011). Aminoglycoside resistance in multiply antibiotic-resistant *Acinetobacter baumannii* belonging to global clone 2 from Australian hospitals. *J. Antimicrob. Chemother.* 66 1504–1509. 10.1093/jac/dkr163 21586593

[B43] Orlek A. Stoesser N. Anjum M. F. Doumith M. Ellington M. J. Peto T. (2017). Plasmid classification in an era of whole-genome sequencing: application in studies of antibiotic resistance epidemiology. *Front. Microbiol.* 8:182 10.3389/fmicb.2017.00182 PMC5299020 28232822

[B44] Ou H.-Y. Kuang S. N. He X. Molgora B. M. Ewing P. J. Deng Z. (2015). Complete genome sequence of hypervirulent and outbreak-associated *Acinetobacter baumannii* strain LAC-4: epidemiology, resistance genetic determinants and potential virulence factors. *Sci. Rep.* 5:8643 10.1038/srep08643 PMC4345345 25728466

[B45] Peleg A. Y. de Breij A. Adams M. D. Cerqueira G. M. Mocali S. Galardini M. (2012). The success of *Acinetobacter* species; genetic, metabolic and virulence attributes. *PLoS ONE* 7:e46984 10.1371/journal.pone.0046984 PMC3483291 23144699

[B46] Ramírez M. S. Vilacoba E. Stietz M. S. Merkier A. K. Jeric P. Limansky A. S. (2013). Spreading of AbaR-type genomic islands in multidrug resistance *Acinetobacter baumannii* strains belonging to different clonal complexes. *Curr. Microbiol.* 67 9–14. 10.1007/s00284-013-0326-5 23397241

[B47] Recchia G. D. Hall R. M. (1995). Gene cassettes: a new class of mobile element. *Microbiology* 141 3015–3027. 10.1099/13500872-141-12-3015 8574395

[B48] Segal H. Elisha B. G. (1999). Characterization of the *Acinetobacter* plasmid, pRAY, and the identification of regulatory sequences upstream of an *aadB* gene cassette on this plasmid. *Plasmid* 42 60–66. 10413667 10.1006/plas.1999.1403

[B49] Thomas C. M. Thomson N. R. Cerdeño-Tárraga A. M. Brown C. J. Top E. M. Frost L. S. (2017). Annotation of plasmid genes. *Plasmid* 91 61–67. 10.1016/j.plasmid.2017.03.006 28365184

[B50] Val M.-E. Bouvier M. Campos J. Sherratt D. Cornet F. Mazel D. (2005). The single-stranded genome of phage CTX is the form used for integration into the genome of *Vibrio cholerae*. *Mol. Cell* 19 559–566. 10.1016/j.molcel.2005.07.002 16109379

[B51] Wallace L. Daugherty S. C. Nagaraj S. Johnson J. K. Harris A. D. Rasko D. A. (2016). Use of comparative genomics to characterize the diversity of *Acinetobacter baumannii* surveillance isolates in a health care institution. *Antimicrob. Agents Chemother.* 60 5933–5941. 10.1128/AAC.00477-16 27458211 PMC5038335

[B52] World Health Organization (2017). *WHO Publishes List of Bacteria for Which New Antibiotics are Urgently Needed.* Geneva: World Health Organization Available at: http://www.who.int/mediacentre/news/releases/2017/bacteria-antibiotics-needed/en/

[B53] Zimbler D. L. Arivett B. A. Beckett A. C. Menke S. M. Actis L. A. (2013). Functional features of TonB energy transduction systems of *Acinetobacter baumannii*. *Infect. Immun.* 81 3382–3394. 10.1128/IAI.00540-13 23817614 PMC3754232

