# Supplementary figures and images for: Small, Enigmatic Plasmids of the Nosocomial Pathogen, Acinetobacter baumannii: Good, Bad, Who Knows?
Source: Front Microbiol. 2017 Aug 15;8:1547. doi: 10.3389/fmicb.2017.01547 (PMC5559437; doi:10.3389/fmicb.2017.01547)

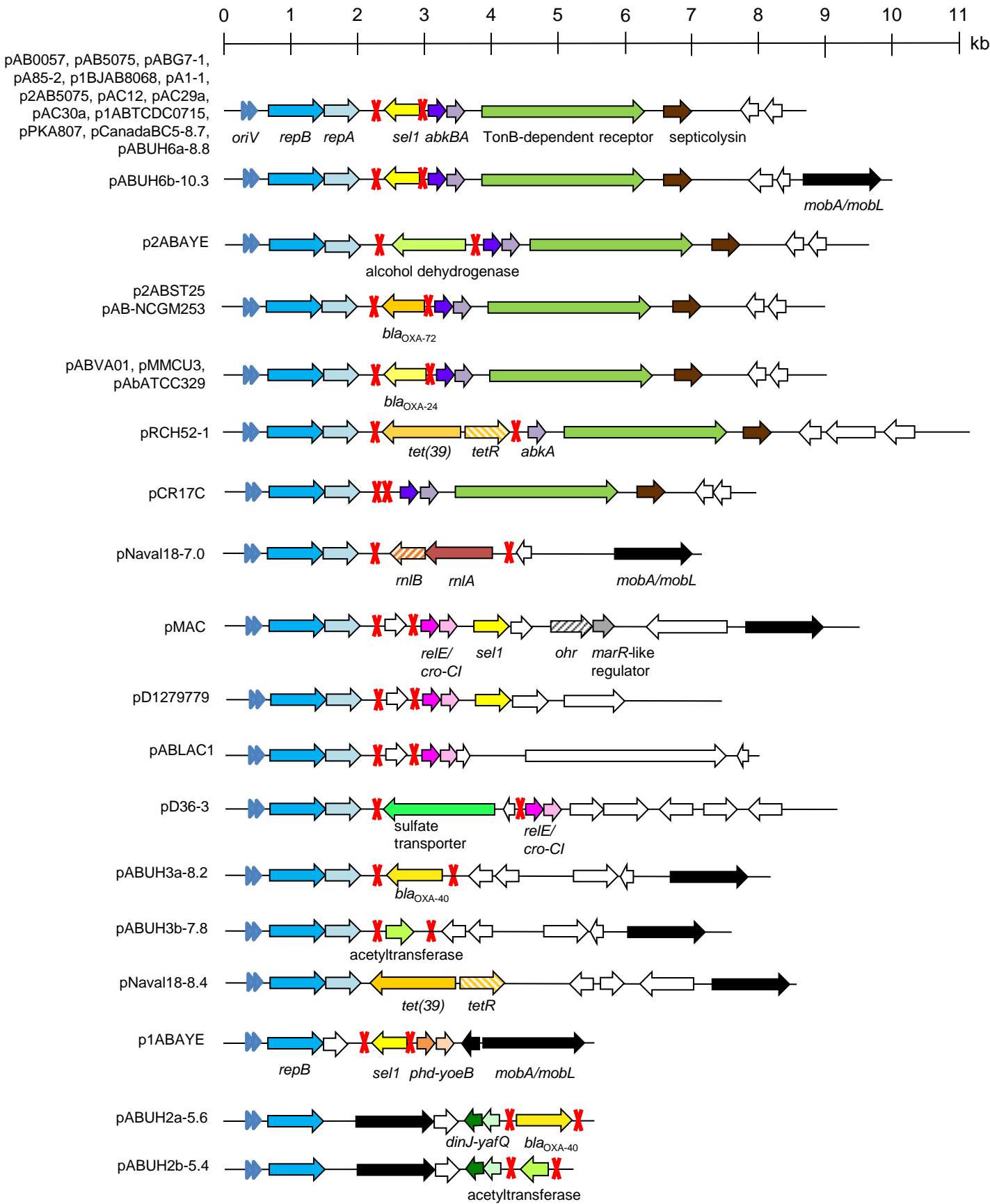

Supplement: FIGURE S1 — Comparative map of the small Acinetobacter plasmids of the Rep-3 superfamily. The repB replicase gene is indicated as a dark blue filled arrow, the putative repA gene is depicted in light blue. Hypothetical open reading frames are shown as unfilled arrows whereas black arrows are for the mobA/mobL mobilization genes. Red crosses indicate the XerC/XerD recombination sites. Filled blue twin-triangles depict the iterons that make up the putative origin of replication, oriV. Accession numbers and further details of the plasmids are as in Supplementary Table S1 with detailed iteron sequences and locations on the respective plasmids in Supplementary Data Sheet 1. [file Image_1.PDF]

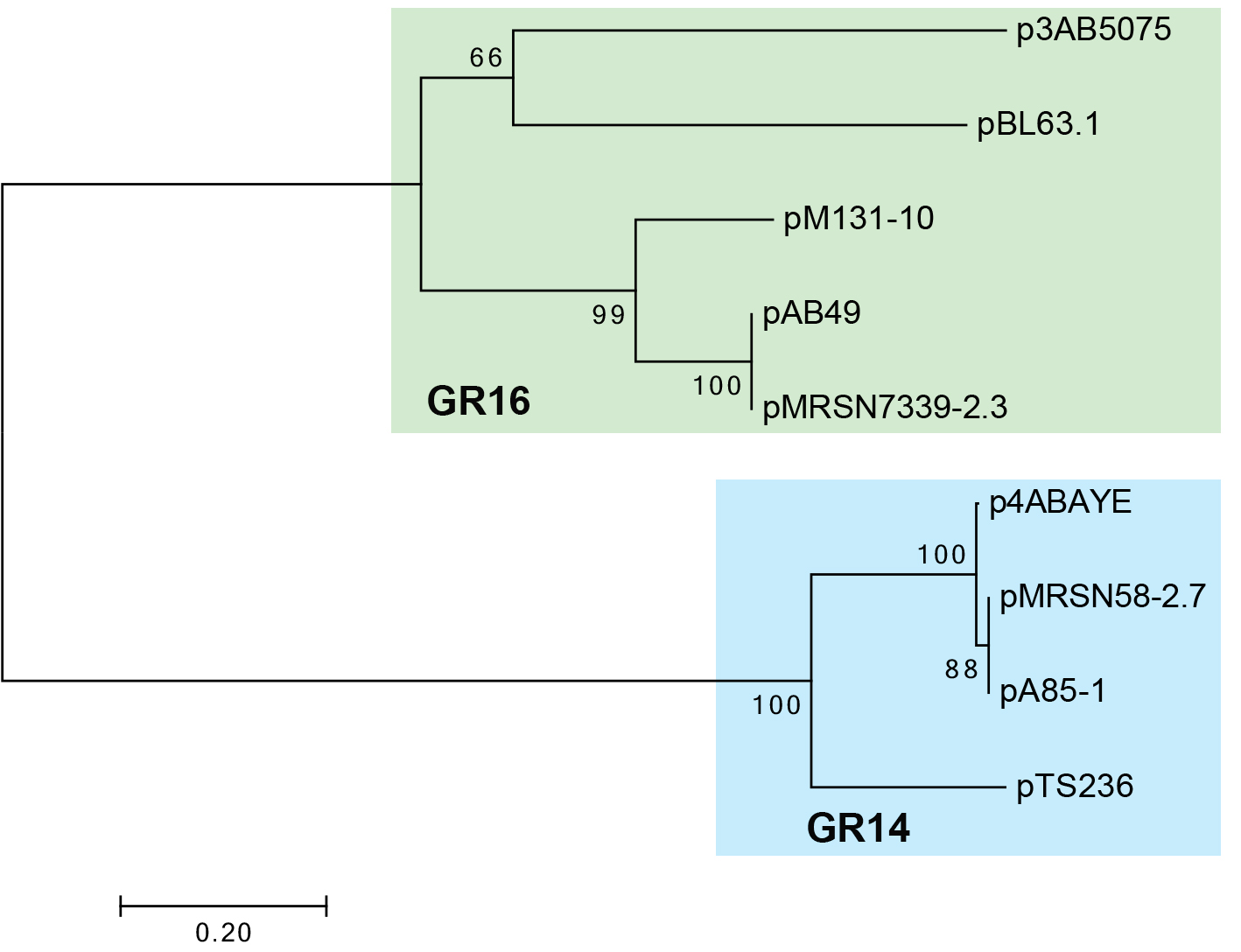

Supplement: FIGURE S2 — Phylogenetic tree of the small Acinetobacter plasmids of the Rep-1 superfamily based on the Rep protein sequences, analyzed and drawn using MEGA7 (Kumar et al., 2016). Protein sequences were aligned using MUSCLE (Edgar, 2004), evolutionary history was inferred using the Neighbor-Joining method and the optimal tree (with the sum of branch length = 3.01508040) is shown. The percentage of replicate trees in which the associated taxa clustered together in the bootstrap test (1000 replicates) is shown next to the branches. The tree is drawn to scale, with branch lengths in the same units as those of the evolutionary distances used to infer the phylogenetic tree. The plasmids were grouped according to the GR classification scheme proposed by Bertini et al. (2010) and indicated here as GR14 and GR16 in different colored boxes. Accession numbers for the plasmids used in the analysis are as follows: p3AB5075 (NZ_CP008709.1), pBL63.1 (NC_006959.1), pM131-10 (NC_025169.1), pAB49 (L77992.1), pMRSN7339-2.3 (NZ_CM003313.1), p4ABAYE (NC_010403.1), pMRSN58-2.7 (NZ_CM003316.1), pA85-1 (NC_025107.1), and pTS236 (NC_016977.1). Note that pBL63.1 was isolated from Bacillus lichineformis and was included in the analysis based on the findings of Guglielmetti et al. (2005). [file Image_2.JPEG]
