# Supplementary material for: Small, Enigmatic Plasmids of the Nosocomial Pathogen, Acinetobacter baumannii: Good, Bad, Who Knows?
Source: Front Microbiol. 2017 Aug 15;8:1547. doi: 10.3389/fmicb.2017.01547 (PMC5559437; doi:10.3389/fmicb.2017.01547)

Supplementary Material

Small, Enigmatic Plasmids of the Nosocomial Pathogen, *Acinetobacter baumannii*: Good, Bad, Who Knows?

Soo Sum Lean[1], Chew Chieng Yeo[2*]

[1]Saw Swee Hock School of Public Health, National University of Singapore, Singapore.

[2]Biomedical Research Centre, Faculty of Medicine, Universiti Sultan Zainal Abidin, Kuala Terengganu, Malaysia.

*** Correspondence:** Chew Chieng Yeo: email: chewchieng@gmail.com

Supplemental File 1. Sequences of the putative replication region of the Rep-3 superfamily *Acinetobacter* plasmids analyzed in this study and grouped according the GR groupings based on the RepB sequences (Bertini et al., 2010; see main text and Figure 1). Iteron sequences are highlighted in grey and indicated by dashed red arrows (--->) above. Partial iteron sequences are highlighted in yellow and indicated by dotted red arrows (...>) above. All iteron sequences were identified using Tandem Repeats Finder (https://tandem.bu.edu/trf/trf.html) followed by manual curation. The *repB* reading frame is highlighted in blue. Other reading frames are highlighted in green. Directions of the respective reading frames are indicated by blue arrows. *** denote a stop codon. Each sequence is numbered according to its GenBank entry. Multiple sequence alignment of the iteron sequences is shown for each GR group with more than one member and when there are differences in the iteron sequences. Alignment of iterons from representative plasmids of all eight groups of Rep-3 *Acinetobacter* plasmids with relation to the RepB phylogenetic tree is indicated at the end of this file (page 40).

GR1 group

p1ABSDF (CU468231.1; 6106 bp)

```
6001 tatttttca aacggaaggc gaatatttct ataaacacat tcaaaacaac ccaacggtcc
6061 atttggaata gacacaagca ccgagcgagt ggctacgagc gattga
   hyp ORF     ** *
   1 acagtatcaa aatttccacc ccgccttttc cctgattaca aagtttcctt ttacacctta
  61 acgagattaa ccgagcaaag cgacggtata acctttgcga aatgaccgag cgtaagcgag
 121 aaaaaataca tgagcgaagc gaattcatag ttgcttttc ttttggattt cccaactaaa
 181 attaaccaaa aactgcccct cgaatcgagc aaagcgagat tcaatagagt acgaacaaag
 241 tgagtaccaa gggcggtttt aagcactccc cctattttta atatttttta ttttttatt
                                                     ---------------------
 301 ttttagtgct cataatacta tgataaatat ataaaaaaat atcaataagt acacctttat
 ---->------------------------>------------------------>---------------
 361 cttgcaataa gtacaccttt atcttgcaat aagtacacct ttatcttgca ataagtacac
 ----------->
 421 ctttatcttg taaaatgaca cttaattaaa taaagtgtac ttataacctt gaaaaaaata
                                                        repB
 481 tgtgtactta tgaagaagga acttgttgtc aaagacaatg cactaataaa tgccagttat
```

Iteron 1 (343..364): caataagtacacctttatcttg (22 nt)
Iteron 2 (365..386): caataagtacacctttatcttg
Iteron 3 (387..408): caataagtacacctttatcttg
Iteron 4 (409..430): caataagtacacctttatcttg

GR2 group

pD36-3 (NZ_CP012955.1; 9276 bp)

```
8101 taatttactg gttctgattc cttctgaatt agccaattga tcgtagaatt ctgctacttg
8161 tggatctaac ctgactgcat aagttttgac tttttcgccc ataaaattaca cctcttatta
                         hyp ORF (AN415_7012)
8221 aaattagatt ttgtacaaaa ttaaatatat ataattcaac taattaaata aaaaataata
8281 agtagattaa gtacaaacct atttgtacag aattaattta ttttaaaaca aaaacttatt
8341 taaatataat gctttttttta cgtacaatca tgagtacata gtttttctta aatttatcaa
8401 tatcttatct aaaaaaagtg ttgtttttat gtacaaccat gagcacagca taaaaaaata
8461 gataattcaa tcacttattc taaaaaaaga ccatttttct gtacacgttt ctgagaccct
8521 aaaaaatcgc tgaaatcctt atcctgtaag gctttgaggg ttgtcatctt tagatgcgtg
8581 gtgtgtgaca aaaaaatccc ggcgtgtgcc ggatccttga ttggaaaatt ggctaaagtg
8641 acgtagggcc ggtgcttggt tttgcatgga aaaaagtatt tatttcttgg tttagaaaaa
8701 cgtaaaaaga tcaatttttt tgtcccttca tccaggttaa aaatttcaac ctaaaatttt
8761 aattatgaaa agcttcacag aaagcattca aatgcgattt aagagccttt acctaaaaag
8821 catagatctc atagcgaaaa acagaaaaca gctcaaaaaa cgcaaatgag agcgaaataa
8881 agagggattt tgactttaga tagatgcata gagcgagtgt ctacgagcga actatcaaaa
8941 tttgcgccta gaccctctga aaaacatttt tttgtcctct ttagcctaag aaagcgtaag
9001 tttcatgcag aaatttgctc ctggatcgag cgtagcgagt aaaaaagttt tatgagcgaa
9061 gcgaattccg agttgctttt gctttttcta aaagtcacgt aagtattaac caagaaattg
9121 ccccgacgaa ctgagcgcaa gcgaagttca atagagttta agcgaagcga aaaccaaggg
9181 caatttttca ttccctgggc ttttaattat tttaaagttt ttaaatgctt ttaaatggct
                                          ----
9241 tgaaagcctt atgaataaag ggttttagct cttata
    -------------------->------------------------>---------------------
   1 tgtccacgtt taccttgcaa tatgtccacg tttaccttgc aatatgtcca cgtttacctt
    --->----------------------->
  61 gcaatatgtc cacgtttacc ttgcattagt acacaaataa tattaacgtg tacttataca
 121 caataaaaaa atagtggcta tgagagattt agttgtaaaa gacaatgcct taatcaacgc
                         repB
 181 aagctataac ttagacttag tagaacaacg tttaattttta ttggctattg ttgaagcaag
```

```
Iteron 1 (9274..19): atatgtccacgtttaccttgca (22 nt)
Iteron 2 (20..41)   : atatgtccacgtttaccttgca
Iteron 3 (42..63)   : atatgtccacgtttaccttgca
Iteron 4 (64..85)   : atatgtccacgtttaccttgca
```

GR2 group

pPKAB07 (CP006964.1; 8805 bp)

```
8341 gtttagaaaa acgtaaaaaa tcaatttttt gtccattcat ccaggttaaa aatttcaacc
             hypothetical ORF (U476_20005)
8401 caaaacttta attatgaaaa gcttcataga acgcgttcaa atgcgattta agagccttta
8461 cctagaaaac atagatctca tagtgaaaaa cacaaaacag ctcaaaaaac gcaaatgaga
8521 gcgaaataaa gaggtatttt gactttagat agatgcatag agcgagtgtc tacgagcgaa
8581 ctatcaaaat ttgcgcctag accctctgaa aaacattttt ttgtcctctt tagcctaaga
8641 aagcgtaagt ttcgtgcaga aatttgctcc tggatcgagc gtagcgagta aaaaagtttt
8701 atgagcgaag cgaattccaa gtttcttttg cttttttta aagtcacgcc agtattaacc
8761 aaaaaattgc cccaacgaac tgagcgaaag cgaagttcaa tagag
   1 tttttttaa agtcacgcca gtattaacca aaaaattgcc ccaacgaact gagcgaaagc
  61 gaagttcaat agagtttgag cgaagcgaaa accaagggca atttttcatt ccctgggctt
 121 ttaattattt taaagttttt aaatactttt aaatggcttg aaagccttat gaataaaggg
                  ------------------------>------------------------>------
 181 ttttagctct tatatgtcca cgtttacctt gcaatatgtc cacgtttacc ttgcaatatg
     ------------------->------------------------>
 241 tccacgttta ccttgcaata tgtccacgtt taccttgcat tagtacacaa ataatattaa
 301 cgtgtactta tacacaataa aaaatagtgg ctatgagaga tttagttgta aaggacaatg
                                        repB
 361 ccttaatcaa cgcaagctat aacttagact tagtagaaca acgtttaatt ttattggcta
```

```
Iteron 1 (192..213): atatgtccacgtttaccttgca (22 nt)
Iteron 2 (214..235): atatgtccacgtttaccttgca
Iteron 3 (236..257): atatgtccacgtttaccttgca
Iteron 4 (258..279): atatgtccacgtttaccttgca
```

pCanada-BC5-8.7 (NZ_AFDN01000003.1; 8731 bp)

```
8641 ttcgtgcaga aatttgctcc tggatcgagc gtagcgagta aaaaagtttt atgagcgaag
8701 cgaattccaa gtttcttttg cttttttta a
   1 agtcacgcca gtattaacca aaaaattgcc ccaacgaact gagcgaaagc gaagttcaat
hyp. ORF
  61 agagtttgag cgaagcgaaa accaagggca atttttcatt ccctgggctt ttaattattt
 121 taaagttttt aaatactttt aaatggcttg aaagccttat gaataaaggg ttttagctct
       ------------------------->------------------------>-----------------
 181 tatatgtcca cgtttacctt gcaatatgtc cacgtttacc ttgcaatatg tccacgttta
    -------->------------------------->
 241 ccttgcaata tgtccacgtt taccttgcat tagtacacaa ataatattaa cgtgtactta
 301 tacacaataa aaaatagtgg ctatgagaga tttagttgta aaggacaatg ccttaatcaa
                               repB
 361 cgcaagctat aacttagact tagtagaaca acgtttaatt ttattggcta ttgttgaagc
```

```
Iteron 1 (182..203): atatgtccacgtttaccttgca (22 nt)
Iteron 2 (204..225): atatgtccacgtttaccttgca
Iteron 3 (226..247): atatgtccacgtttaccttgca
Iteron 4 (248..269): atatgtccacgtttaccttgca
```

GR2 group

pAC30a (CP007578; 8685 bp) (*reverse complement*)

```
tagaaaaacg taaaaaatca atttttgtc cattcatcca ggttaaaaat ttcaacccaa 541
   hypothetical ORF (B856_19010)
aactttaatt atgaaaagct tcatagaacg cgttcaaatg cgatttaaga gcctttacct 481
agaaaacata gatctcatag tgaaaaacac aaaacagctc aaaaaacgca aatgagagcg 421
aaataaagag gtattttgac tttagataga tgcatagagc gagtgtctac gagcgaacta 361
tcaaaatttg cgcctagacc ctctgaaaaa catttttttg tcctctttag cctaagaaag 301
cgtaagtttc gtgcagaaat ttgctcctgg atcgagcgta gcgagtaaaa aagttttatg 241
agcgaagcga attccaagtt tcttttgctt ttttttaaag tcacgccagt attaaccaaa 181
aaattgcccc aacgaactga gcgaaagcga agttcaatag agtttgagcg aagcgaaaac 121
caagggcaat ttttcattcc ctgggctttt aattatttta aagtttttaa atacttttaa 61
                                          ----------------------->
atggcttgaa agccttatga ataaagggtt ttagctctta tatgtccacg tttaccttgc 1
                    ----------------------->
                    tatgt ccacgtttac cttgcattag tacacaaata atattaacgt 8641
gtacttatac acaataaaaa atagtggcta tgagagattt agttgtaaag gacaatgcct 8581
                                 repB
taatcaacgc aagctataac ttagacttag tagaacaacg tttaatttta ttggctattg 8521

Iteron 1 (21..1)     : atatgtccacgtttaccttgc- (21 nt)
Iteron 2 (8685..8665): -tatgtccacgtttaccttgca
```

Note: Incomplete iterons for pAC30a could be due to possible sequencing and/or assembly errors.

pAC29a (NZ_CP008850.1; 8737 bp)

```
7321 aaagcgtaag tttcgtgcag aaatttgctc ctggatcgag cgtagcgagt aaaaaagttt
7381 tatgagcgaa gcgaattcca agtttctttt gctttttttt aaagtcacgc cagtattaac
                hypothetical ORF (BL01_RS18575)
7441 caaaaaattg ccccaacgaa ctgagcgaaa gcgaagttca atagagtttg agcgaagcga
7501 aaaccaaggg caatttttca ttccctgggc ttttaattat tttaaagttt ttaaatactt
                                                --------------------
7561 ttaaatggct tgaaagcctt atgaataaag ggttttagct cttatatgtc cacgtttacc
    ----->----------------------->------------------------>-------------
7621 ttgcaatatg tccacgttta ccttgcaata tgtccacgtt taccttgcaa tatgtccacg
    ------------->
7681 tttaccttgc attagtacac aaataatatt aacgtgtact tatacacaat aaaaaatagt
7741 ggctatgaga gatttagttg taaaggacaa tgccttaatc aacgcaagct ataacttaga
         repB

Iteron 1 (7604..7625): atatgtccacgtttaccttgca (22 nt)
Iteron 2 (7626..7647): atatgtccacgtttaccttgca
Iteron 3 (7648..7669): atatgtccacgtttaccttgca
Iteron 4 (7670..7691): atatgtccacgtttaccttgca
```

GR2 group

pAC12 (CP007550.1; 8731 bp)

```
5041 aaaaagtatt tatttccttg tttagaaaaa cgtaaaaaat caatttttttg tccattcatc
                                  hypothetical ORF (A478_18540)
5101 caggttaaaa atttcaaccc aaaactttaa ttatgaaaag cttcatagaa cgcgttcaaa
5161 tgcgatttaa gagcctttac ctagaaaaca tagatctcat agtgaaaaac acaaaacagc
5221 tcaaaaaacg caaatgagag cgaaataaag aggtattttg actttagata gatgcataga
5281 gcgagtgtct acgagcgaac tatcaaaatt tgcgcctaga ccctctgaaa aacatttttt
5341 tgtcctcttt agcctaagaa agcgtaagtt tcgtgcagaa atttgctcct ggatcgagcg
5401 tagcgagtaa aaaagtttta tgagcgaagc gaattccaag tttcttttgc ttttttttaa
5461 agtcacgcca gtattaacca aaaaattgcc ccaacgaact gagcgaaagc gaagttcaat
5521 agagtttgag cgaagcgaaa accaagggca atttttcatt ccctgggctt ttaattattt
5581 taaagttttt aaatactttt aaatggcttg aaagccttat gaataaaggg ttttagctct
      ------------------------->------------------------->------------------
5641 tatatgtcca cgtttacctt gcaatatgtc cacgtttacc ttgcaatatg tccacgttta
    -------->------------------------->
5701 ccttgcaata tgtccacgtt taccttgcat tagtacacaa ataatattaa cgtgtactta
5761 tacacaataa aaaatagtgg ctatgagaga tttagttgta aaggacaatg ccttaatcaa
                           repB
```

```
Iteron 1 (5642..5663): atatgtccacgtttaccttgca (22 nt)
Iteron 2 (5664..5685): atatgtccacgtttaccttgca
Iteron 3 (5686..5707): atatgtccacgtttaccttgca
Iteron 4 (5708..5729): atatgtccacgtttaccttgca
```

pAB5075 (NZ_JHUI01000005.1; 8819 bp)

```
 661 aaaaagtttt atgagcgaag cgaattccaa gtttcttttg cttttttttta aagtcacgcc
                             hypothetical ORF (A591_RS19395)
 721 agtattaacc aaaaaattgc cccaacgaac tgagcgaaag cgaagttcaa tagagtttga
 781 gcgaagcgaa aaccaagggc aatttttcat tccctgggct tttaattatt ttaaagtttt
                                                                 ----------
 841 taaatacttt taaatggctt gaaagcctta tgaataaagg gttttagctc ttatatgtcc
    ----------------->------------------------->------------------------->---
 901 acgtttacct tgcaatatgt ccacgtttac cttgcaatat gtccacgttt accttgcaat
    ---------------------->
 961 atgtccacgt ttaccttgca ttagtacaca aataatatta acgtgtactt atacacaata
1021 aaaaatagtg gctatgagag atttagttgt aaaggacaat gccttaatca acgcaagcta
                   repB
```

```
Iteron 1 (893..914): atatgtccacgtttaccttgca (22 nt)
Iteron 2 (915..936): atatgtccacgtttaccttgca
Iteron 3 (937..958): atatgtccacgtttaccttgca
Iteron 4 (959..980): atatgtccacgtttaccttgca
```

GR2 group

pABUH6a-8.8 (NZ_AYEX01000118.1; 8763 bp)

```
8461 cgtagcgagt aaaaaagttt tatgagcgaa gcgaattcca agtttctttt gctttttttt
8521 aaagtcacgc cagtattaac caaaaaattg ccccaacgaa ctgagcgaaa gcgaagttca
hyp. ORF
8581 atagagtttg agcgaagcga aaaccaaggg caatttttca ttccctgggc ttttaattat
8641 tttaaagttt ttaaatactt ttaaatggct tgaaagcctt atgaataaag ggttttagct
                                   ------------------------->...............
8701 cttatatgtc cacgtttacc ttgcaatatg tccacgttta ccttgcaata tgtccacgtt
    ...>
8761 tac
    .................>------------------------>------------------------>
   1 ccacgtttac cttgcaatat gtccacgttt accttgcaat atgtccacgt taccttgca
  61 ttagtacaca aataatatta acgtgtactt atacacaata aaaaatagtg gctatgagag
                                                            repB
 121 atttagttgt aaaggacaat gccttaatca acgcaagcta taacttagac ttagtagaac

Iteron 1 (8726..8747) : atatgtccacgtttaccttgca (22 nt)
Partial 1 (8748..8763): atatgtccacgtttac          } Possible sequence assembly
Partial 2 (1..16)      :       ccacgtttaccttgca    } error?
Iteron 2 (17..38)      : atatgtccacgtttaccttgca
Iteron 3 (39..60)      : atatgtccacgtttaccttgca
```

p2ABST25 (AEPA01000396.1; 8970 bp)

```
241 cgtaagtttc gtgcagaaat ttgctcctgg atcgagcgta gcgagtaaaa aagttttatg
301 agcgaagcga attccaagtt tcttttgctt ttttttaaag tcacgccagt attaaccaaa
                                    hypothetical ORF
361 aaattgcccc aacgaactga gcgaaagcga agttcaatag agtttgagcg aagcgaaaac
421 caagggcaat tttcattcc ctgggctttt aattatttta aagtttttaa atacttttaa
                                                 --------------------------
481 atggcttgaa agccttatga ataaagggtt ttagctctta tatgtccacg tttaccttgc
  ->------------------------->------------------------->-------------------
541 aatatgtcca cgtttacctt gcaatatgtc cacgtttacc ttgcaatatg tccacgttta
  ------->
601 ccttgcatta gtacacaaat aatattaacg tgtacttata cacaataaaa aatagtggct
661 atgagagatt tagttgtaaa ggacaatgcc ttaatcaacg caagctataa cttagactta
        repB

Iteron 1 (520..541): atatgtccacgtttaccttgca (22 nt)
Iteron 2 (542..563): atatgtccacgtttaccttgca
Iteron 3 (564..585): atatgtccacgtttaccttgca
Iteron 4 (586..607): atatgtccacgtttaccttgca
```

GR2 group

pABNCGM-253 (NC_021489.1; 8970 bp) (*reverse complement*)

```
gtcacgccag tattaaccaa aaaattgccc caacgaactg agcgaaagcg aagttcaata 8221
gagtttgagc gaagcgaaaa ccaagggcaa tttttcattc cctgggcttt taattatttt 8161
       hypothetical ORF (D721_p5009)
aaagttttta aatactttta aatggcttga aagccttatg aataaagggt tttagctctt 8101
------------------------>------------------------>-----------------
atatgtccac gtttaccttg caatatgtcc acgtttacct tgcaatatgt ccacgtttac 8041
----->------------------------>
cttgcaatat gtccacgttt accttgcatt agtacacaaa taatattaac gtgtacttat 7981
acacaataaa aaatagtggc tatgagagat ttagttgtaa aggacaatgc cttaatcaac 7921
                       repB
gcaagctata acttagactt agtagaacaa cgtttaattt tattggctat tgttgaagca 7861

Iteron 1 (8100..8079): atatgtccacgtttaccttgca (22 nt)
Iteron 2 (8078..8057): atatgtccacgtttaccttgca
Iteron 3 (8056..8035): atatgtccacgtttaccttgca
Iteron 4 (8034..8013): atatgtccacgtttaccttgca
```

pAB-G7-1 (NC_025110.1; 8731 bp)
pA85-2 (NC_025108.1; 8731 bp)
pA1-1 (NZ_CP010782.1; 8731 bp)
pAB0057 (CP001183.2; 8731 bp)

```
8581 acgaactgag cgaaagcgaa gttcaataga gtttgagcga agcgaaaacc aagggcaatt
8641 tttcattccc tgggctttta attattttaa agtttttaaa tacttttaaa tggcttgaaa
hyp. ORF                                   -----
8701 gccttatgaa taaagggttt tagctcttat a
    --------------------->-------------------------->----------------------
   1 tgtccacgtt taccttgcaa tatgtccacg tttaccttgc aatatgtcca cgtttacctt
    --->-------------------------->
  61 gcaatatgtc cacgtttacc ttgcattagt acacaaataa tattaacgtg tacttataca
 121 caataaaaaa tagtggctat gagagattta gttgtaaagg acaatgcctt aatcaacgca
                              repB
 181 agctataact tagacttagt agaacaacgt ttaattttat tggctattgt tgaagcaagg

Iteron 1 (8729..19): atatgtccacgtttaccttgca (22 nt)
Iteron 2 (20..41)  : atatgtccacgtttaccttgca
Iteron 3 (42..63)  : atatgtccacgtttaccttgca
Iteron 4 (64..85)  : atatgtccacgtttaccttgca
```

GR2 group

p1BJAB8068 (NC_021730.1; 8721 bp)

```
2101 ctcctggatc gagcgtagcg agtaaaaaag ttttatgagc gaagcgaatt ccaagtttct
2161 tttgcttttt tttaaagtca cgccagtatt aaccaaaaaa ttgccccaac gaactgagcg
 hypothetical ORF
(BJAB0868_RS19570)
2221 aaagcgaagt tcaatagagt ttgagcgaag cgaaaaccaa gggcaatttt tcattccctg
2281 ggcttttaat tattttaaag tttttaaata cttttaaatg gcttgaaagc cttatgaata
                         ------------------------>------------------------>
2341 aagggtttta gctcttatat gtccacgttt accttgcaat atgtccacgt ttaccttgca
     ------------------------>
2401 atatgtccac gtttaccttg caattacctt gcattagtac acaaataata ttaacgtgta
2461 cttatacaca ataaaaaata gtggctatga gagatttagt tgtaaaggac aatgccttaa
                                          repB
2521 tcaacgcaag ctataactta gacttagtag aacaacgttt aattttattg gctattgttg

Iteron 1 (2357..2378): atatgtccacgtttaccttgca (22 nt)
Iteron 2 (2379..2400): atatgtccacgtttaccttgca
Iteron 3 (2401..2422): atatgtccacgtttaccttgca
```

p1ABTCDC0715 (CP002523.1; 8731 bp)

```
 901 ctgagcgaaa gcgaagttca atagagtttg agcgaagcga aaaccaaggg caatttttca
                                                      hypothetical ORF
 961 ttccctgggc ttttaattat tttaaagttt ttaaatactt ttaaatggct tgaaagcctt
                              ------------------------>------------------
1021 atgaataaag ggttttagct cttatatgtc cacgtttacc ttgcaatatg tccacgttta
     -------->------------------------>------------------------>
1081 ccttgcaata tgtccacgtt taccttgcaa tatgtccacg tttaccttgc attagtacac
1141 aaataatatt aacgtgtact tatacacaat aaaaaatagt ggctatgaga gatttagttg
                                                    repB
1201 taaaggacaa tgccttaatc aacgcaagct ataacttaga cttagtagaa caacgtttaa

Iteron 1 (1044..1065): atatgtccacgtttaccttgca (22 nt)
Iteron 2 (1066..1087): atatgtccacgtttaccttgca
Iteron 3 (1088..1109): atatgtccacgtttaccttgca
Iteron 4 (1110..1131): atatgtccacgtttaccttgca
```

GR2 group

p2AB5075 (NZ_CP008708.1; 8731 bp)

```
8341 gatcgagcgt agcgagtaaa aaagttttat gagcgaagcg aattccaagt ttcttttgct
8401 ttttttttaaa gtcacgccag tattaaccaa aaaattgccc caacgaactg agcgaaagcg
       hyp. ORF
  (ABUW_RS20060)
8461 aagttcaata gagtttgagc gaagcgaaaa ccaagggcaa tttttcattc cctgggcttt
8521 taattatttt aaagttttta aatactttta aatggcttga aagccttatg aataaagggt
                ------------------------->------------------------->--------
8581 tttagctctt atatgtccac gtttaccttg caatatgtcc acgtttacct tgcaatatgt
     ------------------>------------------------->
8641 ccacgtttac cttgcaatat gtccacgttt accttgcatt agtacacaaa taatattaac
8701 gtgtacttat acacaataaa aaatagtggc t
   1 atgagagatt tagttgtaaa ggacaatgcc ttaatcaacg caagctataa cttagactta
             repB
  61 gtagaacaac gtttaatttt attggctatt gttgaagcaa gggaaagtgg gaaagggatt

Iteron 1 (8591..8612): atatgtccacgtttaccttgca (22 nt)
Iteron 2 (8613..8634): atatgtccacgtttaccttgca
Iteron 3 (8635..8656): atatgtccacgtttaccttgca
Iteron 4 (8657..8678): atatgtccacgtttaccttgca
```

p2ABAYE (NC_010402.1; 9661 bp)

```
361 aaattgcccc aacgaactga gcgaaagcga agttcaatag agtttgagcg aagcgaaaac
421 caagggcaat ttttcattcc ctgggctttt aattatttta aagtttttaa atacttttaa
hypothetical ORF
 (ABAYE_RS19480)                                       ------------------------>
481 atggcttgaa agccttatga ataaagggtt ttagctctta tatgtccacg tttaccttgc
    ->------------------------->------------------------->------------------
541 aatatgtcca cgtttacctt gcaatatgtc cacgtttacc ttgcaatatg tccacgttta
    -------->
601 ccttgcatta gtacacaaat aatattaacg gtgtacttat acacaataaa aaatagtggc
661 tatgagagat ttagttgtaa aggacaatgc cttaatcaac gcaagctata acttagactt
            repB

Iteron 1 (520..541): atatgtccacgtttaccttgca (22 nt)
Iteron 2 (542..563): atatgtccacgtttaccttgca
Iteron 3 (564..585): atatgtccacgtttaccttgca
Iteron 4 (586..607): atatgtccacgtttaccttgca
```

GR2 group

pAB2 (CP000523.1; 11302 bp)

```
10981 gtcacgcaag tattaaccaa aaaattgccc caacgaactg agcgaaagcg aagttcaata
11041 gagtttgagc gaagcgaaaa ccaagggcaa tttttcattc cctgggcttt taattatttt
                 hypothetical ORF
11101 aaatttttta aatgctttta aatggcttga aagccttatg aataaagggt tttagctctt
      ----------------------->----------------------->------------------
11161 atatgtccac gtttaccttg caatatgtcc acgtttacct tgcaatatgt ccacgtttac
     ------>----------------------->
11221 cttgcaatat gtccacgttt accttgcatt agtacacaaa taatattaac gtgtacttat
11281 acacaataaa aaaatagtgg ct
    1 atgagagatt tagttgtaaa agacaatgcc ttaatcaacg caagctataa cttagactta
      repB
   61 gtagaacaac gtttaatttt attggctatt gttgaagcaa gggaaagtgg gaaagggatt
```

Iteron 1 (11161..11182): atatgtccacgtttaccttgca (22 nt)
Iteron 2 (11183..11204): atatgtccacgtttaccttgca
Iteron 3 (11205..11226): atatgtccacgtttaccttgca
Iteron 4 (11227..11248): atatgtccacgtttaccttgca

pABNA1 (GQ338082.1; 6095 bp, partial)

```
  1 gtcacgccag tattaaccaa aaaattgccc caacgaactg agcgaaagcg aagttcaata
 61 gagtttgagc gaagcgaaaa ccaagggcaa tttttcattc cctgggcttt taattatttt
121 aaagttttta aatactttta aatggcttga aagccttatg aataaagggt tttagctctt
    ----------------------->----------------------->-------------------
181 atatgtccac gtttaccttg caatatgtcc acgtttacct tgcaatatgt ccacgtttac
    ------>----------------------->----------------------->
241 cttgcaatat gtccacgttt accttgcaat atgtccacgt ttaccttgca ttagtacaca
301 aataatatta acgtgtactt atacacaata aaaaatagtg gctatgagag atttagttgt
                                                  repB
361 aaaggacaat gccttaatca acgcaagcta taacttagac ttagtagaac aacgtttaat
```

Iteron 1 (181..202): atatgtccacgtttaccttgca (22 nt)
Iteron 2 (203..224): atatgtccacgtttaccttgca
Iteron 3 (225..246): atatgtccacgtttaccttgca
Iteron 4 (247..268): atatgtccacgtttaccttgca
Iteron 5 (269..290): atatgtccacgtttaccttgca

GR2 group

pACICU1 (NC_010605.1; 28279 bp)

```
  1 gaattccaag tttcttttgc ttttttttaa agtcacgcca gtattaacca aaaaattgcc
                        hypothetical ORF
 61 ccaacgaact gagcgaaagc gaagttcaat agagtttgag cgaagcgaaa accaagggca
121 atttttcatt ccctgggctt ttaattattt taaagttttt aaatactttt aaatggcttg
                                     ------------------------>---------
181 aaagccttat gaataaaggg ttttagctct tatatgtcca cgtttacctt gcaatatgtc
 ---------------->------------------------>------------------------>--
241 cacgtttacc ttgcaatatg tccacgttta ccttgcaata tgtccacgtt taccttgcaa
 ------------------------>
301 tatgtccacg tttaccttgc attagtacac aaataatatt aacgtgtact tatacacaat
361 aaaaaatagt ggctatgaga gatttagttg taaaggacaa tgccttaatc aacgcaagct
                         repB
421 ataacttaga cttagtagaa caacgtttaa ttttattggc tattgttgaa gcaagggaaa

Iteron 1 (212..233): atatgtccacgtttaccttgca (22 nt)
Iteron 2 (234..255): atatgtccacgtttaccttgca
Iteron 3 (256..277): atatgtccacgtttaccttgca
Iteron 4 (278..299): atatgtccacgtttaccttgca
Iteron 5 (300..321): atatgtccacgtttaccttgca
```

Note: all GR2 plasmids that were analyzed in this study contained identical 22 nt iteron sequences. Majority of the iterons consisted of four direct repeats with the exception of p1BJAB8068 which consisted of three direct repeats, and pABNA1 and pACICU1 which contained five direct repeats. Plasmid pAC30a contained two 21 nt incomplete iteron sequences whereas pABUH61-8.8 contained three direct repeats and two partial iteron sequences; these could be due to sequencing assembly errors (see pages 5 and 7).

GR3 group

p736 (GU978996.1; 1065 bp, partial)

```
    ----------------------->----------------------->-------------------
  1 taaaacgagg tttaccttgc attaaaacga ggtttacctt gcattaaaac gaggtttacc
   ----->----------------------->
 61 ttgcattaaa acgaggttta ccttgcatta agcgagttaa taatataacc tcgtcttatt

121 aaattaaaaa cataatcttt tcatatgaaa acagaactaa tagttaaaga taatgcctta
                               repB
181 attaatgcca gttataacct tgatctagtg gagcaacggt taattctttt agcgatcctt

Iteron 1 (1..21) : -taaaacgaggtttaccttgca (21 nt)
Iteron 2 (22..43): ttaaaacgaggtttaccttgca (22 nt)
Iteron 3 (44..65): ttaaaacgaggtttaccttgca
Iteron 4 (66..87): ttaaaacgaggtttaccttgca
```

p203 (GU978997.1; 1068 bp, partial)

```
    ----------------------->----------------------->--------------------
  1 taaaacgagg tttaccttgc attaaaacga ggtttacctt gcattaaaac gaggtttacc
   ----->----------------------->
 61 ttgcattaaa acgaggttta ccttgcatta agcgagttta tgatatagcc tcgtcttatt

121 gaattaaaaa tataaccttt tcatatgaaa acagaactaa tagttaaaga taatgcatta
                               repB
181 attaatgcca gttataacct tgatctagtc gagcaacggt taattcttct agctatcgtt

Iteron 1 (1..21)  : -taaaacgaggtttaccttgca (21 nt)
Iteron 2 (22..43) : ttaaaacgaggtttaccttgca (22 nt)
Iteron 3 (44..65) : ttaaaacgaggtttaccttgca
Iteron 4 (66..87) : ttaaaacgaggtttaccttgca
```

pRCH52-1 (KT346360.1; 11164 bp)

```
10081 tgacttttttc gcccataaat tacacctctt attaaaatta gattttgtac aaaattaaat
          hyp ORF
10141 atatataatt caactaatta aataaaaaat aataagtaga ttacgtacaa acctatttgt
10201 acagaattaa tttattttaa aacaaaaact tatttaaata taatgctttt tttatgtaca
10261 atcatgagta catagttttt cttaaattta tcaatatctt atctaaaaaa agtgttgttt
10321 tgatgtacaa ccatgagcac agcataaaaa atagataatt caatcactta ttctaaaaga
10381 agaccatttt tctgtacacg tttctgagac cctaaaaaat cgctgaaatc cttatcctgt
10441 aaggctttga ggggtgtcat cgttagatgc gtggtgtgtg acaaaaaaat cccggcatgt
10501 gccggatctt ggattaaaaa attgactaaa gtgacgtagg gctggtgctt ggttttgcat
10561 ggaaaaagta tttatttcct ggtttagaaa aatgtaaaaa gatcaatttt tttgtccctt
10621 catctaggtt caaaattcca acctaaaact ttgagtatga aaagcttcac agaaagcgtt
10681 caaatgtgat ttaagagccg ttacctaaaa aacatagctt tttatagtga aaaatagaaa
10741 acagctcaaa aaatgcaaat gagagcgaaa tagaggggggg ttgactctag atagattcat
10801 agagcgagtg tctacgagcg aactatcaaa atttgcgcct agactttctg aaaaacagtt
10861 tttgccctct ttagcctaag aaagcgtaag tttcatgtag aaatttgctt ctcgaccgag
10921 cgtagcgagt aaaaaagttt tatgagcgaa gcgaattccg agttgctttt gctttttctt
10981 aaagtcacgt aagtattaac caaaaaattg ccccgacgaa ctgagcgaaa gcgaagttca
11041 atagagtttg agcgaagcga aaaccaaggg caatttttcc ttccctgggc ttttaagtat
11101 tttaaagttt ttaaatgctt ttagatggtc taaaaacctt tatttataac actcttagag
          --
11161 atta
      ---------------------->---------------------->--------------------
    1 taaaacgagg tttaccttgc attaaaacga ggtttacctt gcattaaaac gaggtttacc
      ---->----------------------->
   61 ttgcattaaa acgaggttta ccttgcatta agcgagttaa taatatagcc tcgtcttatt

  121 aaattaaaaa cataatcttt tcatatgaaa acagaactaa tagttaaaga taatgctcta
                                 repB
  181 attaatgcca gttataacct agatttagtc gagcaaaggt taattcttct agcgatactt


Iteron 1 (11164..21)   : ataaaacgaggtttaccttgca (22 nt)
Iteron 2 (22..43)      : ttaaaacgaggtttaccttgca
Iteron 3 (44..65)      : ttaaaacgaggtttaccttgca
Iteron 4 (66..87)      : ttaaaacgaggtttaccttgca
```

GR3 group

pABLAC1 (NZ_CP007713.1; 8006 bp)

```
601 gcttttgctt tttcttaaag tcacgcaagt attaaccaaa aaattgcccc gacgaactga
661 gcgaaagcga agttcaatag agtttgagcg aagcgaaaac caagggcaat ttttcatgac
                                  hypothetical ORF   ***
721 ctgagctttt aaatattttt aaatctttta aatgctttta gacatgctga aagcctttat
                          ----------------------->-------------------
781 ttataaaatt cttagagact ataaaacgag gtttaccttg cattaaaacg aggtttacct
   ---->------------------------>------------------------>
841 tgcattaaaa cgaggtttac cttgcattaa aacgaggttt accttgcatt aagcgagttt
901 atgatatagc ctcgtcttat tgaattaaaa atataacctt ttcatatgaa aacagaacta
                                                            repB
961 atagttaaag ataatgcatt aattaatgcc agttataacc ttgatctagt cgagcaacgg

Iteron 1 (801..822): ataaaacgaggtttaccttgca (22 nt)
Iteron 2 (823..844): ttaaaacgaggtttaccttgca
Iteron 3 (845..866): ttaaaacgaggtttaccttgca
Iteron 4 (867..888): ttaaaacgaggtttaccttgca
```

pOIFC143-6.2 (NZ_AFDL01000007.1; 6241 bp)

```
1921 cagtttttgt tcattcatcc aggttaaaaa tttcaaccta aaactttaat tatgaaaagc
hypothetical ORF
1981 ttcacagaaa gcattcaaat gcgatttaag agcctttatc taaaaaacat agatctatag
2041 cgaaaaacag aaaacagctc aaaaaacgca aaagagagtg aagtaaagag atgttttgac
2101 tttagatagc tgcatagagc gagtgtctac gagcgaacta tcaaaatttg cgcctagact
2161 ctctgaaaaa catttttttg ccctctttag cctaagaaag cttaagtttc atgcagaaat
2221 ttgctcctgg accgagcgta gcgagaaaaa aagctcatga gcgaagcgaa ttccgagttg
2281 cttttgcttt ttcttaaagt cacgcaagta ttaaccaaaa aattgccccg acgaactgag
2341 cgaaagcgaa gttcaataga gtttgagcga agcgaaaacc aagggcaatt tttcatgacc
2401 tgagctttta aatattttta aatcttttaa atgtttttag acatgctgaa agcctttatt
                           ------------------------->--------------------
2461 tataaaattc ttagagacta taaaacgagg tttaccttgc attaaaacga ggtttacctt
     --->-------------------------->------------------------>
2521 gcattaaaac gaggtttacc ttgcattaaa acgaggttta ccttgcatta agcgagttta
2581 tgatatagcc tcgtcttatt gaattaaaaa tataaccttt tcatatgaaa acagaactaa
                                                        repB
2641 tagttaaaga taatgcatta attaatgcca gttataacct tgatctagtc gagcaacggt

Iteron 1 (2480..2501): ataaaacgaggtttaccttgca (22 nt)
Iteron 2 (2502..2523): ttaaaacgaggtttaccttgca
Iteron 3 (2524..2545): ttaaaacgaggtttaccttgca
Iteron 4 (2546..2567): ttaaaacgaggtttaccttgca
```

GR3 group

pD1279779 (NC_020525.1; 7416 bp)

```
6841 aaaacgtaaa aagatcagtt tttgttcatt catccaggtt aaaaatttca acctaaaact
                         hypothetical ORF
6901 ttaattatga aaagcttcac agaaagcatt caaatgcgat ttaagagcct ttatctaaaa
6961 aacatagatc ttatagcgaa aaacagaaaa cagctcaaaa aacgcaaaag agagtgaagt
7021 aaagagatgt tttgacttta gatagctgca tagagcgagt gtctacgagc gaactatcaa
7081 aatttgcgtc tagactctct gaaaaacatt tttttgccct ctttagccta agaaagctta
7141 attttcatgc agaaatttgc tcctggaccg agcgtagcga gaaaaaaagc tcatgagcga
7201 agcgaattcc gagttgcttt tgctttttct taaagtcacg caagtattaa ccaaaaaatt
7261 gccccgacga actgagcgaa agcgaagttc aatagagttt gagcgaagcg aaaaccaagg
7321 gcaatttttc atgacctgag cttttaaata tttttaaatc ttttaaatgc ttttagacat
                                                --
7381 gctgaaagcc tttatttata aaattcttag agacta
    ------------------------>------------------------>--------------------
   1 taaaacgagg tttaccttgc attaaaacga ggtttacctt gcattaaaac gaggtttacc
    ----->------------------------>
  61 ttgcattaaa acgaggttta ccttgcatta agcgagttta tgatatagcc tcgtcttatt
 121 gaattaaaaa tataaccttt tcatatgaaa acagaactaa tagttaaaga taatgcatta
                               repB
 181 attaatgcca gttataacct tgatctagtc gagcaacggt taattcttct agctatcgtt

Iteron 1 (7416..21)   : ataaaacgaggtttaccttgca (22 nt)
Iteron 2 (22..43)     : ttaaaacgaggtttaccttgca
Iteron 3 (44..65)     : ttaaaacgaggtttaccttgca
Iteron 4 (66..87)     : ttaaaacgaggtttaccttgca
```

GR8 group

p11921 (GU979000.1; 1103 bp, partial)

```
    ----------------------->----------------------->------------------
  1 taggtttatc gacccataaa attaggttta tcgacccata aaattaggtt tatcgactca
    ----->---------------------->----------------------->........>
 61 taaaactagg tttatcgacc ttaaaactag gtttatcgac cttaaaacta ggttgattta
         ..........>
121 caagtccatt aaaatagtct attgtacgac ctagtttaat tgtaaattta ttattagaaa
                                                                    └─> repB
181 tgagtgaatt aatcgtaaag gataatgctt taattcaggc tagctatact ttagatacag
241 ttgaacaaag actgatctta ttagctattg ctgaagctcg agaaacagga catgggataa

Iteron 1 (1..22)    :  taggtttatcgacccataaaat (22 nt)
Iteron 2 (23..44)   :  taggtttatcgacccataaaat
Iteron 3 (45..66)   :  taggtttatcgactcataaaac
Iteron 4 (67..87)   :  taggtttatcgac-cttaaaac (21 nt)
Iteron 5 (88..108)  :  taggtttatcgac-cttaaaac
Partial 1 (109..116):  taggttgat-------------
Partial 2 (126..135):  -------------ccattaaaat
```

GR8 group

pNaval18-8.4 (NZ_AFDA02000008; 8422 bp) *(reverse complement)*

```
accgattcaa tccgaacagc caaagccaaa atctgatgat tatgattacg gtggtccggg 4021
tttttaaaat tatctggttt tattcaggct ttacagagcg agtgtctacg agcgaacaga 3961
      *** mobA/mobL
cccaattaaa attttcgcca tgctttttct ctgattacag gctccccttt gctgctaaga 3901
ctgaggtaca gagcatcccg aagggtgcga acagattcaa tttgcttcta ggtcgagcga 3841
agcgagcata aaaaaatgat gagcgaagcg aattccgagt tgcttttgct ttttctaaaa 3781
ctcacgaaaa tattaaccaa aaaattgccc ctcgaatcga gcgaagcgag attcaataga 3721
gtttgagcgt agcgaaaacc aagggcaatt tttcatggtt taggctttta attattttta 3661
                                              ---------------
atcttttaaa tgcttttaag taagctgtaa tcctttatac acaaggggttt cataactaat 3601
---->----------------------->----------------------->--------------
aaaactaggt ttatcgaccc ataaaactag gtttatcgac ccataaaact aggtttatcg 3541
------------>---------------------->----------------------->
acccataaaa ctaggtttat cgaccttaaa actaggttta tcgacctcaa atcaagttga 3481
tttacaaatc cattaaaata gtctattgta caacctagtt taattataaa tttattatta 3421
gaaatgagtg aattaatcgt aaaggataat gctttaattc aagctagcta taccctagat 3361
   └──► repB
acagttgaac aaaggctgat cctattagcc attgctgaag ctcgagaaac aggacacggg 3301
```

```
Iteron 1 (3615..3596): --ggtttcataactaataaaac (20 nt)
Iteron 2 (3595..3574): taggtttatcgacccataaaac (22 nt)
Iteron 3 (3573..3552): taggtttatcgacccataaaac
Iteron 4 (3551..3530): taggtttatcgacccataaaac
Iteron 5 (3529..3509): taggtttatcgacc-ttaaaac (21 nt)
Iteron 6 (3508..3488): taggtttatcgacc-tcaaatc
```

GR8 group

pMAC (NC_006877; 9540 bp)

```
8941 tacaacctaa agctgatgat tatgattacg gtggccctgg ttttaagct cagttggttt
                                           mobA     ***
9001 tatccaaagt ttacagagcg agtgtctacg agcgaacaga cacaattaaa atttttgcca
9061 tgctttttcc ctgattacaa actccccttt gctgctgaga ctgagataca gagcatcccg
9121 gagggtgcga acagatttaa tttgcatcta ggtcgagcga agcgagtata aaaaacttat
9181 gagcgaagcg aattccgagt tgcttttgct tttttgaagt tatcacgaga ttaacccaaa
9241 aattgcccct tcgaactgag cgtaagcgaa gttcaataga gtttgagcgt agcgaaaaca
9301 tagggcaatt tttcattcat tgggctttta attattttta atcttttaaa tgcttttaaa
                                -----------------------&gt;-----------------
9361 taagctgtaa acctttattt ataatagttt catagttaat aaaactaggt ttatcgaccc
     -------&gt;-------------------------&gt;-------------------------&gt;-----------
9421 ataaaactag gtttatcgac ccataaaact aggtttatcg acccataaaa ctaggtttat
     -------------&gt;----------------------&gt;
9481 cgaccttaaa gctaggttta tcgacccata attaagttaa tttacaactc aattgaaata
   1 gtctattgta caacctagtt taattataaa tttattatta gaaatgagtg aattaatcgt
                                                        repB
  61 aaaggataat gctctaattc aagcaagtta taccttagat acagttgaac aaagactgat
```

```
Iteron 1 (9385..9405): tagtttcatag-ttaataaaac (21 nt)
Iteron 2 (9406..9427): taggtttatcgacccataaaac (22 nt)
Iteron 3 (9428..9449): taggtttatcgacccataaaac
Iteron 4 (9450..9471): taggtttatcgacccataaaac
Iteron 5 (9472..9492): taggtttatcgacc-ttaaagc (21 nt)
Iteron 6 (9493..9511): taggtttatcgacccataa--- (19 nt)
```

Note: Iteron sequence annotated as ataaaactaggtttatcgacc (9399..9485) in GenBank entry for pMAC

pA21 (GU979001.1; 1120 bp, partial)

```
    .....&gt;-----------------------&gt;-----------------------&gt; -----------
  1 taaaactagg tttatcgacc cttaaaacta ggtttatcga cccttaaaac taggtttatc
   -------------&gt;----------------------&gt;----------------------&gt;
 61 gacccttaaa actaggttta tcgaccttaa aactaggttt atcgaccctt aattaagtta
121 atttacaact caattaaaat agtctattgt acaacctagt ttaattataa atttattatt
181 agaaatgagt gaattaatcg taaaggataa tgctctaatt caagcaagtt ataccttaga
          repB
```

```
Partial (1..6)     :  ----------------taaaac (6 nt)
Iteron 1 (7..28)   :  taggtttatcgacccttaaaac (22 nt)
Iteron 2 (29..50)  :  taggtttatcgacccttaaaac
Iteron 3 (51..72)  :  taggtttatcgacccttaaaac
Iteron 4 (73..93)  :  taggtttatcgacc-ttaaaac (21 nt)
Iteron 5 (94..112):   taggtttatcgacccttaa--- (19 nt)
```

GR8 group

Multiple sequence alignment of GR8 iterons:

p11921

```
Iteron 1 (1..22)     :  taggtttatcgacccataaaat
Iteron 2 (23..44)    :  taggtttatcgacccataaaat
Iteron 3 (45..66)    :  taggtttatcgactcataaaac
Iteron 4 (67..87)    :  taggtttatcgac-cttaaaac
Iteron 5 (88..108)   :  taggtttatcgac-cttaaaac
Partial 1 (109..116):  taggttgat-------------
Partial 2 (126..135):  ------------ccattaaaat
```

pNaval18-8.4

```
Iteron 1 (3615..3596): --ggtttcataactaataaaac
Iteron 2 (3595..3574): taggtttatcgacccataaaac
Iteron 3 (3573..3552): taggtttatcgacccataaaac
Iteron 4 (3551..3530): taggtttatcgacccataaaac
Iteron 5 (3529..3509): taggtttatcgacc-ttaaaac
Iteron 6 (3508..3488): taggtttatcgacc-tcaaatc
```

pMAC

```
Iteron 1 (9385..9405): tagtttcatag-ttaataaaac
Iteron 2 (9406..9427): taggtttatcgacccataaaac
Iteron 3 (9428..9449): taggtttatcgacccataaaac
Iteron 4 (9450..9471): taggtttatcgacccataaaac
Iteron 5 (9472..9492): taggtttatcgacc-ttaaagc
Iteron 6 (9493..9511): taggtttatcgacccataa---
```

pA21

```
Partial (1..6)     :    ----------------taaaac
Iteron 1 (7..28)   :    taggtttatcgacccttaaaac
Iteron 2 (29..50)  :    taggtttatcgacccttaaaac
Iteron 3 (51..72)  :    taggtttatcgaccctaaaac
Iteron 4 (73..93)  :    taggtttatcgacc-ttaaaac
Iteron 5 (94..112):    taggtttatcgaccctta a---
```

GR11 group

p1ABAYE (NC_010401.1; 5644 bp)

```
5581 acacgcctta gcaatcccat ttaagcgcaa aaaaacgggg gttttagcga cttttgatag
               ** *  MobA/MobL
5641 aaat
   1  acagtaaaac tatagttgat aaaaataaat cgcttaaaac gcaaatgaga gccttttagc
  61  gagtgtctac gagtgacaca acgaaaggtt gcttattccc cctctgaaaa accgtttttt
 121  attgaattac tggggacagt gattagcgag tgtctacgag cgaagtattg atgcttttgc
 181  tcgtcaaaaa gcatgagcat agcgaatgca ttattcatgt tttgcttttt gaatccttga
 241  tcatatatgc aaaaaactgc ccccacgaag cgagtgaaac gagcttcaat agagtacgag
 301  ctttagcgag taccaagggc agttttaata actcactttg gaaataaatt cccttttatt
 361  cttttaaaaa gcttttaaaa gcttttagac cctctgaaaa ccttatgcag caatagtttc
             ----------------------->----------------------->
 421  aagcgttaaa agggtacaaa tagcatgata aagggtacaa atagcatgat tttatcgttt
        -----------------------> ----------------------->-------------------
 481  aaaagggtac aaatagcatg attaaagggt acaaatagca tgataaaggg tacaaataaa
     ------>                        ......>                  ------------------------
 541  ttattgtacc cttatgttta ttggtgtaca ttgttttctc ataagggtac aaaaatcatg
     -->
 601  ttcatttttt ataaggtttg atatctgaat gaataaagaa aatagttatg ataaatctta
                                        repB
```

```
Iteron 1 (428..449): aaagggtacaaatagcatgat (21 nt)
Iteron 2 (450..470): aaagggtacaaatagcatgat
Iteron 3 (482..502): aaagggtacaaatagcatgat
Iteron 4 (504..524): aaagggtacaaatagcatgat
Iteron 5 (525..538): aaagggtacaaataaattatt
Partial (564..570) : ---gtgtaca-----------
Iteron 6 (582..602): taagggtacaaaaatcatgtt
```

pABUH2a-5.6 (NZ_AYFZ01000080.1; 5636 bp)

```
4921 tctggttcta ttcgtaatga aattacttat aagtcgctag aaaatcttgg aatcatttaa
                                                  blaOXA-24         ***
4981 taatttctaa aattaacata atacaccttta tacgaaatac gttataattc cacttagaaa
                 XerD                XerC
5041 tcaatgctgt agtttctagt ttatagccca atcgagcccg tttgggcttt ttttatgatt
5101 tttcatgttc cacgaatatt ctttaatcaa tgttccacgg ttttgattag atattttaat
5161 cattccagta agggccgagc gagtgtctac gagcgaccac ctggcattaa aatttagccc
5221 ctgccttttc tctgattaca agctcccttt tgcttcgtaa cgaaagatac agagcatccc
5281 gaatgggtgc gaactgtatt agtcaaattc tagacgagcg aagcgagcat aaagactact
5341 gagcgaagcg aaggcatagt ctcttttta ttaaatcctc aattcatatt tgaaaaaaac
5401 agcccctcaa ccgagcgaaa gcgaggttaa aacgtccgag ctttagcgag ggcatagggc
5461 tgttttattt tctgtatttt cctttttat cttttaaaaa gcttttaaaa gcttttagac
                                                  -------------------->
5521 ccccttctag cccatatcta gcaaggcttt cagactttaa aaaagtactt ttaccggaca
       -------------------->                  -------------------->
5581 accaagtact tttaccggac aaaacgcatt taaaaaagta cttttaccgg acttaa
     -------------------->                      ..........>      ......
   1 aaagtacttt taccggacat taaggtacac aaatttagat gtacttttttt taaaaaagta
.>                                                ..........>      ......
  61 caaaaataat agtcaaattt taagcaataa tatactatac aggtacatat ccgaaaaagt
....>
 121 acttaatagg cattggagtt atatggacga aaataaaaaa acgtatccac cttcttgggt
                                  repB
```

```
Iteron 1 (5562..5580): aaagtacttttaccggaca (19 nt)
Iteron 2 (5583..5601): caagtacttttaccggaca
Iteron 3 (5615..5633): aaagtacttttaccggact
Iteron 4 (1..19)     : aaagtacttttaccggaca
Partial 1 (38..48)   : gatgtactttt--------
Partial 2 (55..65)   : aaagtac------------
Partial 3 (100..110) : caggtacatat--------
Partial 4 (116..126) : aaagtactt----------
```

GR11 group

pABUH2b-5.4 (NZ_AYFH01000057.1; 5355 bp)

```
5041 tcaaaaagag tatgcattac atcaagctcc aaatattaaa tagttaataa gcatatacat
acetyltransferase
5101 atttttattt ttttgcgagt aagtgtttga gttttaataa tattacatat aagtaaattt
5161 tattttagcg aataaacaga aagaacaaat gtatttaagt ttgagcctta cgaaaagctg
5221 tattatgaaa aatagctaaa attaacataa tacaccttat acgaaatacg ttataattcc
                           XerC                     XerD
5281 acttagaaat caatgctgta gtttctagtt tatagcccaa tcgagcccgt ttgggctttt
5341 tttatgattt ttcat
   1 cttagaaatc aatgctgtag tttctagttt atagcccaat cgagcccgtt tgggcttttt
  61 ttatgatttt tcatgttcca cgaatattct ttaatcaatg ttccacggtt ttgattagat
 121 attttaatca ttccagtaag ggccgagcga gtgtctacga gcgaccacct ggcattaaaa
 181 tttagcccct gccttttctc tgattacaag ctcccttttg cttcgtaacg aaagatacag
 241 agcatcccga atgggtgcga actgtattag tcaaattcta gacgagcgaa gcgagcataa
 301 agactactga gcgaagcgaa ggcatagtct ctttttttatt aaatcctcaa ttcatatttg
 361 aaaaaaacag cccctcaacc gagcgaaagc gaggttaaaa cgtccgagct ttagcgaggg
 421 catagggctg ttttattttc tgtattttcc ttttttatct tttaaaaagc ttttaaaagc
                                                              ------------
 481 ttttagaccc ccttctagcc catatctagc aaggctttca gactttaaaa aagtactttt
    -------->  ------------------->                        ------------------
 541 accggacaac caagtacttt taccggacaa aacgcattta aaaaagtact tttaccggac
    ->    -------------------->  ........>               ..........>     ...
 601 ttaaaaagta cttttaccgg acattaaggt acacaaattt agatgtactt tttttaaaaa
    ....>                                         ..........>          ..
 661 agtacaaaaa taatagtcaa attttaagca ataatatact atacaggtac atatccgaaa
    .......>
 721 aagtacttaa taggcattgg agttatatgg acgaaaataa aaaaacgtat ccaccttctt
                              repB

Iteron 1 (530..548):  aaagtacttttaccggaca (19 nt)
Iteron 2 (551..569):  caagtacttttaccggaca
Iteron 3 (583..601):  aaagtacttttaccggact
Iteron 4 (605..623):  aaagtacttttaccggaca
Partial 1 (625..637): taggtac------------
Partial 2 (643..651): gatgtactttt--------
Partial 3 (659..669): aaagtac------------
Partial 4 (704..714): caggtacatat--------
Partial 3 (720..730): aaagtactt----------
```

Multiple sequence alignment of GR11 iterons:

```
                  Iteron 1 (428..449) :  aaagggtacaaatagcatgat
                  Iteron 2 (450..470) :  aaagggtacaaatagcatgat
                  Iteron 3 (482..502) :  aaagggtacaaatagcatgat
p1ABAYE           Iteron 4 (504..524) :  aaagggtacaaatagcatgat
                  Iteron 5 (525..538) :  aaagggtacaaataaattatt
                  Partial  (564..570) :  ---gtgtaca-----------
                  Iteron 6 (582..602) :  taagggtacaaaaatcatgtt

                  Iteron 1 (5564..5580): aaa--gtacttttaccggaca
                  Iteron 2 (5583..5601): caa--gtacttttaccggaca
                  Iteron 3 (5615..5633): aaa--gtacttttaccggact
pABUH2a-5.6       Iteron 4 (1..19)     : aaa--gtacttttaccggaca
                  Partial 1 (38..48)   : gat--gtactttt--------
                  Partial 2 (55..65)   : aaa--gtac------------
                  Partial 3 (100..110) : cag--gtacatat--------
                  Partial 3 (116..126) : aaa--gtactt----------

                  Iteron 1 (531..548)  : aaa--gtacttttaccggaca
                  Iteron 2 (552..569)  : caa--gtacttttaccggaca
                  Iteron 3 (584..601)  : aaa--gtacttttaccggact
                  Iteron 4 (606..623)  : aaa--gtacttttaccggaca
pABUH2b-5.4       Partial 1 (626..637) : tag--gtac------------
                  Partial 2 (643..651) : gat--gtactttt--------
                  Partial 3 (660..669) : aaa--gtac------------
                  Partial 4 (705..714) : cag--gtacatat--------
                  Partial 3 (721..730) : aaa--gtactt----------
```

Note: The three plasmids that made up the GR11 group contained the most varied of iteron sequences within each group of plasmids. The iteron sequence appeared to be made up of just a core GTAC(A/T)(A/T)(A/T)T sequence. It is possible that p1ABAYE may form a separate group (or sub-group) from the pABUH2a and pABUH2b plasmids. The availability of sequences from other plasmids within this group would validate this possibility.

GR12 group

pNaval81-13 (NZ_AFDB02000005.1; 12634 bp)

```
11341 tctagtctga ctgcataagt tttgactttt tcgcccataa attacatctc ttataaaaat
                            hypothetical ORF
11401 taaagtttgt acaagattaa atcaagataa ttcaataatt tagatcaaaa taataaataa
11461 attacataca aatctatttg tacaaaatta atttattata aaacaaaaac ttatttaaat
11521 ataatgcttt ttttatgtac aactatgagt acatattttt tattagcttt atcaatatct
11581 tatctaaaaa aagcgttgtt ttgatgtaca agtatgagca cagcataaaa aataaataat
11641 tcaattactt attctaaaaa aagactattt ttctgtacac gtttctaaga cattgaaaaa
11701 ttgctgaaac ctttatcctg taaggttttg agggctgtca tctttagatg cgtggtgtgt
11761 gacaaaaaaa tcacggcatg tgccggatcc ttgattaaaa aattggctaa agtgacgtag
11821 ggctggtgct tggtttttac atggaaaaaa gtatttattt cctgatttag aaaaacgtaa
11881 aaaaatcgat tttttgtcca ttcatccaag ttagaaattt caacccaaaa ctttaattgt
11941 gaaaagcctc atagaaagcg ttcaaatgcg atttaagagc ctttatctaa aaaacataga
12001 tcttcatagg gaaaacagaa aacagctcaa aaaacgcaaa tgagagcgaa ataaagaggg
12061 gttttgagtc tagatagatt catagagcga gtgtctacga gcgaactatc aaaagttgcg
12121 cctagaccct ctgaaaaaca ttttttgccc tctttagctt aagaaagcgt aagtttcatg
12181 cagaaatttg cttctcgacc gagcatagcg agtaaaaaag ttttatgagc gtagcgaatt
12241 ccgagttgct ttttgctttt tttgaaagtc acgtaagtat ttaccaaaaa attgccccga
12301 cgaactgagc gaaagcgaag ttcaatagag tttgagcgaa gcgaaaacca agggcaattt
12361 ttcaatctac tctttttaaa tatattttaa atcttttaaa tcttttaggc tatctgaaag

12421 gctttaacag aaaggctttc agctactaac tatgagggat tgactaccaa ctatgaggga

12481 ttgactacca actatgaggg attgactact taactatgag ggattgacta ctaactatga

12541 gagaattata ttactcatag ttaatttctt gaaataagaa tgatgagtac atctaaaaaa
                                                           repB
12601 gaattggtag ttaaatcaaa tcaagtaatt gagg
```

```
Iteron 1 (12443..12463): ctac-taactatgagggattga (21 nt)
Iteron 2 (12464..12484): ctac-caactatgagggattga
Iteron 3 (12485..12505): ctac-caactatgagggattga
Iteron 4 (12506..12527): ctacttaactatgagggattga (22 nt)
Iteron 5 (12528..12548): ctac-taactatgagagaatta
```

pNaval17-13 (NZ_AFDO01000021.1; 12636 bp)

```
7381 tgactgcata agttttgact ttttcgccca taaattacat ctcttataaa aattaaagtt
                hypothetical ORF
7441 tgtacaagat taaatcaaga taattcaata atttagatca aaataataaa taaattacat
7501 acaaatctat ttgtacaaaa ttaatttatt ataaaacaaa aacttattta aatataatgc
7561 tttttttatg tacaactatg agtacatatt ttttattagc tttatcaata tcttatctaa
7621 aaaaagcgtt gttttgatgt acaagtatga gcacagcata aaaaataaat aattcaatta
7681 cttattctaa aaaaagacta tttttctgta cacgtttcta agacattgaa aaattgctga
7741 aacctttatc ctgtaaggtt ttgagggctg tcatctttag atgcgtggtg tgtgacaaaa
7801 aaatcacggc atgtgccgga tccttgatta aaaaattggc taaagtgacg tagggctggt
7861 gcttggtttt tacatggaaa aaagtattta tttcctgatt tagaaaaacg taaaaaaaat
7921 cgattttttg tccattcatc caagttagaa atttcaaccc aaaactttaa ttgtgaaaag
7981 cctcatagaa agcgttcaaa tgcgatttaa gagcctttat ctaaaaaaca tagatcttca
8041 tagggaaaac agaaaacagc tcaaaaaacg caaatgagag cgaaataaag aggggttttg
8101 agtctagata gattcataga gcgagtgtct acgagcgaac tatcaaaagt tgcgcctaga
8161 ccctctgaaa aacatttttt gccctcttta gcttaagaaa gcgtaagttt catgcagaaa
8221 tttgcttctc gaccgagcat agcgagtaaa aaagttttat gagcgtagcg aattccgagt
8281 tgctttttgc tttttttgaa agtcacgtaa gtatttacca aaaaattgcc ccgacgaact
8341 gagcgaaagc gaagttcaat agagtttgag cgaagcgaaa accaagggca atttttcaat
8401 ctactctttt taaatatatt ttaaatcttt taaatctttt aggctatctg aaaggcttta
                                  ----------------------->----------------------->---
8461 acagaaaggc tttcagctac taactatgag ggattgacta ccaactatga gggattgact
     -------------------->------------------------->---------------------
8521 accaactatg agggattgac tacttaacta tgagggattg actactaact atgagagaat
     ->
8581 tatattactc atagttaatt tcttgaaata agaatgatga gtacatctaa aaaagaattg
                                        repB
8641 gtagttaaat caaatcaagt aattgaggct tcatatcaac tgagctcaac agaacaacgg
```

```
Iteron 1 (8477..8497): ctac-taactatgagggattga (21 nt)
Iteron 2 (8498..8518): ctac-caactatgagggattga
Iteron 3 (8519..8539): ctac-caactatgagggattga
Iteron 4 (8540..8561): ctacttaactatgagggattga (22 nt)
Iteron 5 (8562..8582): ctac-taactatgagagaatta
```

GR12 group

pXBB1-3 (CP010354.1; 11643 bp)

```
7981 gtcgtgggca aatgagccag aacccgagaa aaaacccaag aaggacaatg gtttcgatct
8041 atccatgtaa aaacatgtcg tagaatcgca cagaattgcg aattttggat tttttatga
hyp. ORF     ***
8101 aaaatgaccc taggtagcta aaaataatta acgatcttaa atcgaaaaat aatagcaatt
8161 agcgagtgtc tacgagcgac atgacaaaaa tttgcttgcc ttttctctga ttacgatctt
8221 cctatccata taacacggtc agaaaagagc atcccgaatg ggtgcgaacg aataatatga
8281 aaattttgtt cgagcgaagc gagtaaagaa gctttatgag cgaagcgaat tccgagttgc
8341 ttttgatctt gcttttgctt tttctcaaat tcacgaaaat atgaaccaaa aaattgcccc
8401 gtcgaatcga gcgaaagcga gattcaatag agtttgagcg tagcgaaaac caagggcaat
8461 ttttaaattt gctcttttta aatatatttt aaatctttta aatcttttag gtggcttgaa
                                      ----------------------->------------------
8521 agccatgtat atcaatgctt tcagctttta actatgaggg attgactact aactatgagg
     ------>----------------------->------------------------>--------------
8581 gattgactac taactatgag ggattgacta ctaaactatg agggattgac tactaactat
     ----------->
8641 gagagaatta tattactcat agttaggttt atagaaatag aattgatgag tacatctaaa
                                                          repB
8701 aaagaattag tagttaaatc aaatcaagta attgaggctt catatcaatt aagctctaca

Iteron 1 (8545..8565): ctttt-aactatgagggattga (21 nt)
Iteron 2 (8566..8586): ctact-aactatgagggattga
Iteron 3 (8587..8607): ctact-aactatgagggattga
Iteron 4 (8608..8629): ctactaaactatgagggattga (22 nt)
Iteron 5 (8630..8650): ctact-aactatgagagaatta
```

pMMCU1 (NC_013056.1; 8771 bp)

```
7321 tttatgatgg tccgggtttt taaaaatgga tctagatcag aaagtacaaa gcgagtgtct
                 mobA/mobL    ***
7381 acgagcgaca tcaccaaaat tagcgcctcg actttctgaa aaaccgcttt ttaaggctgt
7441 atagcctaaa aaagcataaa tcagatgcta aaactggctt ccagaccgag cgaagcgagc
7501 aaaaagcttt atgagcgaag cgaattccga gttgcttttg ctcttgcctt tacttttttct
7561 caaattcacg aaggaattaa cagaaaaatt gccccctgaa tcgagcgaaa gcgagattca
7621 atagagtttg agcgtagcga aaaccacggg caattttttc aaacgtcttt tttaattatt
7681 tttatatctt ttaaatcttt tctttgagct gtatccctta tggagcaagc ctttcaaagt
       ----------------->----------------------->----------------------->
7741 ttaactatga cggattgact actaactatg acggattgac tactaactat gacggattga
     ----------------------->---------------------->
7801 ctacttaact atgacggatt gactactaac tatgacaagt atatagttgt catatataaa
7861 agaaaatcaa tggttttatt ttggataaga atcaaattgt taaatcaaat caagttatag
                              repB
7921 aagcttccta tcaattaagt gctgtagaac agcgtatcgt cttggcagct atttcacgta

Iteron 1 (7742..7758): -----taactatgacggattga (17 nt)
Iteron 2 (7759..7779): ctac-taactatgacggattga (21 nt)
Iteron 3 (7780..7800): ctac-taactatgacggattga
Iteron 4 (7801..7822): ctacttaactatgacggattga (22 nt)
Iteron 5 (7823..7843): ctac-taactatgacaagtata
```

Highlighted in dark blue: annotated as *oriV* in GenBank entry

GR12 group

pAB02 (AY228470.1; partial sequence – 4162 bp) (*reverse complement*)

```
tggcagctga agggcctgtt actaccttaa atacaatagt attaactgca caatctgatg 121
agttagggtc tgagctgtta ggcaaaagtc taaatgttag taatcagttt attgatacta 61
gtaaacttaa acagcgttcg acaactctag gggatgcctt gggtacggaa taaggaattc 1
                          hypothetical ORF (ORF6)      ***
                                                 ga attccgagtt gcttttgctc 4141
ttgcctttac tttttctcaa attcacgaag gaattaacag aaaaattgcc ccctgaatcg 4081
agcgaaagcg agattcaata gagtttgagc gtagcgaaaa ccacgggcaa ttttttcaaa 4021
cgtctttttt aattattttt atatctttta aatcttttct ttgagctgta tcccttatgg 3961
                  ------------------>----------------------->-----
agcaagcctt tcaaagttta actatgacgg attgactact aactatgacg gattgactac 3901
----------------->----------------------->----------------------->
taactatgac ggattgacta cttaactatg acggattgac tactaactat gacaagtata 3841
tagttgtcat atataaaaga aaatcaatgg ttttattttg gataagaatc aaattgttaa 3781
      repB
```

```
Iteron 1 (3942..3926): -----taactatgacggattga (17 nt)
Iteron 2 (3925..3905): ctac-taactatgacggattga (21 nt)
Iteron 3 (3904..3884): ctac-taactatgacggattga
Iteron 4 (3883..3862): ctacttaactatgacggattga (22 nt)
Iteron 5 (3861..3841): ctac-taactatgacaagtata
```

Highlighted in dark blue: annotated as *oriV* in GenBank entry

pABIR (EU294228.1; 29823 bp)

```
29521 aaataaaacc caagttaaaa tgctctcaca tgcgatttca gccacatttt tacttaagac
29581 atagaatcca ctattgaaat gagtaatcag gtcaaaaaac gcaaatgagc gctaaataga
hyp ORF***
29641 ggctattttt attctaatcg ggatgaatag agcgagtgtc tacgagcgaa gcaacaaaat
29701 tcgcacctcg actctctgaa aaacagcttt ttaggctgtg aagcctaaat aagcgtaaat
29761 cacatgcaga aaattgagcc tagatcgagc gtagcgagta aaaaagcttt atgagcgaag
29821 cga
    1 attccgagtt gcttttgctc ttgcctttgc ttttctcaa agtcacaaca gaatgatcta
   61 aaaaattgcc ccacgaatcg agcgaaagcg agattcaata gagtttgagc gtagcgaaaa
  121 ccaagggcaa ttttttcaaa cgtcttttta attattttta tatcttttaa atcttttcat
                                                  ----------------------->-------
  181 tgagctgtat cccttatgga gcaagccttt caacgtctaa ctatgacgga ttgactacta
 -----------------> ----------------------->------------------------->---
  241 actatgacgg attgactact aactatgacg gattgactac ttaactatga cggattgact
 --------------------->
  301 accaaatatg acaagtatat atttgtcata tataaaagaa aatcaatggt tttatttgg
                                                                  repB
  361 ataagaatca aattgttaaa tcaaatcaag ttatagaagc ttcctatcaa ttaagtgctg
```

```
Iteron 1 (214..234): cgtc-taactatgacggattga (21 nt)
Iteron 2 (235..255): ctac-taactatgacggattga
Iteron 3 (256..276): ctac-taactatgacggattga
Iteron 4 (277..298): ctacttaactatgacggattga (22 nt)
Iteron 5 (299..319): ctac-caaatatgacaagtata
```

Highlighted in dark blue: annotated as *oriV* in GenBank entry

GR12 group

p2ABSDF (CU468232.1; 25014 bp)

```
24601 agtttctata tgataaaaaa acctataaaa accatatcta gcaaaggttt gagggttatc
           *** hypothetical ORF
24661 atctttagat gcgtggtgtg tgacaaaaaa atcccggcat gtgccggatt ctggattaga
24721 aaattggcta aagtgacgta ggactggtgc ttggttttac atggaaaaaa gtatttattt
24781 tctggtttat aaaaacgtaa aaagatcagt ttttgttcat tcatccaggt taaaaatttc
                                  hypothetical ORF
24841 aacctaaaac tttaattatg aaaagcttca cagaaagcat tcaaatgcga tttaagagcc
24901 tttatctaaa aaacatagat ctatagcgaa aaacagaaaa cagctcaaaa aacgcaaaag
24961 agagtgaagt aaagagatgt tttgacttta gatagctgca tagagcgagt gtct
    1 acgagcgaac tatcaaaatt tgcgcctaga ctctctgaaa aacatttttt tgccctcttt
   61 agcctaagaa agcgtaagtt tcatgcagaa atttgctcct ggatcgagcg tagcgagtaa
  121 aaaagtttta tgagcgaagc gaattccaag tttcttttgc ttttttttaaa atcacgccag
  181 tattaaccaa aaaattgccc caacgaactg agcgaaagcg aagttcaata gagtttgagc
  241 gaagcgaaaa ccaagggcaa tttttcaatt tgctcttttt aaatatattt taaatatttt
                                                      --------------
  301 aaatattttc agtcccctgt atgccttata cagtaagggt tttaaagctt aactatgacg
 ------>.......>----------------------->----------------------->------
  361 gattgaggat tgactactaa ctatgacgga ttgactacta actatgacgg attgactact
      ------------------>----------------------->
  421 taactatgac ggattgacta ctaactatga caagtatata tttgtcatat attaaaaatt

  481 aatggtttta ttttgaataa aaatcacgtt gtaaaatcaa atcaagtaat agaagcctca
                  repB
  541 tatcaattaa gtgccgtaga gcaacgaatt gtcttagctg ccatttcacg gattccaaaa
```

```
Iteron 1 (350..366)   : -----taactatgacggattga (17 nt)
Partial 1 (367..373)  : ----------------ggattga (7 nt)
Iteron 2 (374..394)   : ctac-taactatgacggattga (21 nt)
Iteron 3 (395..415)   : ctac-taactatgacggattga
Iteron 4 (416..437)   : ctacttaactatgacggattga (22 nt)
Iteron 5 (438..458)   : ctac-taactatgacaagtata
```

pABUH3a-8.2 (AYFH01000048.1; 8190 bp)

```
2101 ttcaaaacgt gaagaccaag aaaaacagca aaaaaatgaa cccaaaaaac cagatcttga
2161 taatgatcct gactacatgc cttggtaacc cccatttaaa cgcaaaaaaa tggggttttt
                          mobA/mobL      ***
2221 agcgatgttt tggtggagta tatggtttca tagccaaatt taaaaagtct cttaaatcga
2281 aaatgagagc atttagcgag tgtctacgag cgacacaaag aaaattcgcc tatccccccc
2341 cctctgaaaa acagcttttt aggctgtgaa gtctaaataa gcgtaaatca catgcagaaa
2401 attgagccta gaccgagcgt agcgagtaaa aaagctctat gagcgaagcg aattccgagt
2461 tgcttttgat cttgctttta ctttttctaa attcactgga acattgacca aaaaattgcc
2521 ccgacgaatc gagcgaaagc gagattcaat agagtttgag cgtagcgaaa accaagggca
2581 atttttcaaa tgccttttta atatatttta aatcttttaa atcttttcag ttgtctctgt
                                 ----------------------->------------------
2641 gccttatata gcaaaggttt cagcgtttaa atatgacgga ttgactacta actatgacgg
     ---->----------------------->----------------------->--------------
2701 attgactact aactatgacg gattgactac ttaactatga cggattgact actaactatg
     --------->                                                   repB
2761 acaagtatat atttgtcata tattagaaaa atcaatggtt ttgttttgaa taaaaatcac
```

```
Iteron 1 (2664..2684): cgtt-taaatatgacggattga (21 nt)
Iteron 2 (2685..2705): ctac-taactatgacggattga
Iteron 3 (2706..2726): ctac-taactatgacggattga
Iteron 4 (2727..2748): ctacttaactatgacggattga (22 nt)
Iteron 5 (2749..2769): ctac-taactatgacaagtata
Annotated repB (2758..3687)
```

pABUH3b-7.8 (AYFZ01000083.1; 7819 bp)

```
5521 aaaaaccaga tcttgataat gatcctgact acatgccttg gtaacccccca tttaaacgca
                                         mobA/mobL    ***
5581 aaaaaatggg gtttttagcg atgttttggt ggagtatatg gtttcatagc caaatttaaa
5641 aagtctctta aatcgaaaat gagagcattt agcgagtgtc tacgagcgac acaaagaaaa
5701 ttcgcctatc cccccccctc tgaaaaacag ctttttaggc tgtgaagtct aaataagcgt
5761 aaatcacatg cagaaaattg agcctagacc gagcgtagcg agtaaaaaag ctctatgagc
5821 gaagcgaatt ccgagttgct tttgatcttg cttttacttt ttctaaattc actggaacat
5881 tgaccaaaaa attgccccga cgaatcgagc gaaagcgaga ttcaatagag tttgagcgta
5941 gcgaaaacca agggcaattt ttcaaatgcc tttttaatat attttaaatc ttttaaatct
                                                  ----------------------->
6001 tttcagttgt ctctgtgcct tatatagcaa aggtttcagc gtttaaatat gacggattga
     ----------------------->----------------------->--------------------
6061 ctactaacta tgacggattg actactaact atgacggatt gactacttaa ctatgacgga
    ---->----------------------->
6121 ttgactacta actatgacaa gtatatattt gtcatatatt agaaaaatca atggttttgt
                  repB
6181 tttgaataaa aatcacgttg taaaatcaaa tcaagtaata gaagcatcgt atcaattaag
```

```
Iteron 1 (6040..6060): cgtt-taaatatgacggattga (21 nt)
Iteron 2 (6061..6081): ctac-taactatgacggattga
Iteron 3 (6082..6102): ctac-taactatgacggattga
Iteron 4 (6103..6124): ctacttaactatgacggattga (22 nt)
Iteron 5 (6125..6145): ctac-taactatgacaagtata
Annotated repB (6134..7063)
```

GR12 group

Multiple sequence alignment of GR12 iterons:

```
              Iteron 1 (12443..12463): ctac-taa-ctatgagggattga
              Iteron 2 (12464..12484): ctac-caa-ctatgagggattga
pNaval81-13   Iteron 3 (12485..12505): ctac-caa-ctatgagggattga
              Iteron 4 (12506..12527): ctacttaa-ctatgagggattga
              Iteron 5 (12528..12548): ctac-taa-ctatgagagaatta

              Iteron 1 (8477..8497) :  ctac-taa-ctatgagggattga
              Iteron 2 (8498..8518) :  ctac-caa-ctatgagggattga
pNaval17-13   Iteron 3 (8519..8539) :  ctac-caa-ctatgagggattga
              Iteron 4 (8540..8561) :  ctacttaa-ctatgagggattga
              Iteron 5 (8562..8582) :  ctac-taa-ctatgagagaatta

              Iteron 1 (8545..8565) :  cttt-taa-ctatgagggattga
              Iteron 2 (8566..8586) :  ctac-taa-ctatgagggattga
pXBB1-3       Iteron 3 (8587..8607) :  ctac-taa-ctatgagggattga
              Iteron 4 (8608..8629) :  ctac-taaactatgagggattga
              Iteron 5 (8630..8650) :  ctac-taa-ctatgagagaatta

              Iteron 1 (7742..7758) :  -----taa-ctatgacggattga
              Iteron 2 (7759..7779) :  ctac-taa-ctatgacggattga
pMMCU1        Iteron 3 (7780..7800) :  ctac-taa-ctatgacggattga
              Iteron 4 (7801..7822) :  ctacttaa-ctatgacggattga
              Iteron 5 (7823..7843) :  ctac-taa-ctatgacaagtata

              Iteron 1 (3942..3926) :  -----taa-ctatgacggattga
              Iteron 2 (3925..3905) :  ctac-taa-ctatgacggattga
pAB02         Iteron 3 (3904..3884) :  ctac-taa-ctatgacggattga
              Iteron 4 (3883..3862) :  ctacttaa-ctatgacggattga
              Iteron 5 (3861..3841) :  ctac-taa-ctatgacaagtata

              Iteron 1 (214..234)   :  cgtc-taa-ctatgacggattga
              Iteron 2 (235..255)   :  ctac-taa-ctatgacggattga
pABIR         Iteron 3 (256..276)   :  ctac-taa-ctatgacggattga
              Iteron 4 (277..298)   :  ctacttaa-ctatgacggattga
              Iteron 5 (299..319)   :  ctac-caa-atatgacaagtata

              Iteron 1 (350..366)   :  -----taa-ctatgacggattga
              Partial  (367..373)   :  ----------------ggattga
p2ABSDF       Iteron 2 (374..394)   :  ctac-taa-ctatgacggattga
              Iteron 3 (395..415)   :  ctac-taa-ctatgacggattga
              Iteron 4 (416..437)   :  ctacttaa-ctatgacggattga
              Iteron 5 (438..458)   :  ctac-taa-ctatgacaagtata

              Iteron 1 (2664..2684):   cgtt-taa-atatgacggattga
              Iteron 2 (2685..2705):   ctac-taa-ctatgacggattga
pABUH3a-8.2   Iteron 3 (2706..2726):   ctac-taa-ctatgacggattga
              Iteron 4 (2727..2748):   ctacttaa-ctatgacggattga
              Iteron 5 (2749..2769):   ctac-taa-ctatgacaagtata

              Iteron 1 (6040..6060):   cgtt-taa-atatgacggattga
              Iteron 2 (6061..6081):   ctac-taa-ctatgacggattga
pABUH3b-7.8   Iteron 3 (6082..6102):   ctac-taa-ctatgacggattga
              Iteron 4 (6103..6124):   ctacttaa-ctatgacggattga
              Iteron 5 (6125..6145):   ctac-taa-ctatgacaagtata
```

GR17 group

pAB1 (CP000522.1; 13408 bp)

```
12421 tttcggcttt ttctgttcag tcatttttct atttccagta cattttggct atgccaaaag
      LysR-type (A1S_3470)
12481 ctccgcagga caagatgcca cgttgagctg aaagcgaata agtcgggtaa tttatataac
12541 caaggcttgc catgttatat aacattacac atcttgcctg tatcaccctc atcctgtatt
12601 gcacttaatc gcatataatc gcacacaaaa aatactgaag gcgtataaaa tcttataatt
12661 gcacttaatt gcacttaatt gcacttaatc gcacaaaaca gaaagacaag gaaatcaaaa
12721 tgatgacatg acatagcgca aaaaaattca tctcctaata aaaacatcaa aagttaaaaa
12781 gatagatcct aaccatgaat ccaataaaat agaacaagac catatttgtt caaaatatat
12841 agctctaaaa tgcattgaat tacgattaca actaaattca caaaaaaaag tacagaacga
12901 gtgtctacga gtgaaataat tgaaatgcgt gcttagaccc tctaaaaaac tgcttttttta
12961 ggctatttag cctaagaagc ataattcaaa tgtagaaata agttcctaga cgagcgtaag
13021 cgagcaaaaa agttctatga gcgaagcgaa ttcatagttg cttttgcctt tgcttttttc
13081 aaattcactc cgggataagt taaaaattgc ccccagaatc gagcgagagc gagattcaat
13141 agagtttgag cgaagcgaaa accaagggca atttttcctg tcttggactt ttaattattt
13201 ttaatagttt ttaaatactt ttagatcagc tgtaaccatt tgtttataat gattttagag
        ----------------------->----------------------->----------------
13261 aatataagac tacaatttct ttgttataag actacaattt ctttgttata agactacaat
     -------->----------------------->
13321 ttctttgtta taagactaca atttctttgc atttgactac attaaatgct aatgtaattt
13381 taataacact tataatcctt gtagtctt
    1 atgaagaatg acttagtcgt aaaagataat gctttaatta atgccagcta taatctcgaa
          repB
   61 gtaacagaac aacgactgat cctattatca attataagag caagagaaac aggccaagga

Iteron 1 (13263..13284): tataagactacaatttctttgt (22 nt)
Iteron 2 (13285..13306): tataagactacaatttctttgt
Iteron 3 (13307..13328): tataagactacaatttctttgt
Iteron 4 (13329..13250): tataagactacaatttctttgc
```

GR20 group

pCR17C (NZ_HG977529.1; 8047 bp)

```
361 aaattgcccc aacgaactga gcgaaagcga agttcaatag agtttgagcg aagcgaaaac
421 caagggccat ttttcattcc ctgggctttt aattattttt aatcttttaa atgcttttag
hyp. ORF                                                 -------------------------
481 acatgctgaa agccttaatt tacaacactt tcaaaggcta tatgacacca tttaccttgc
  ->------------------------->------------------------->-------------------
541 aatatgacac cgtttacctt gcaatatgac accgtttacc ttgcaatatg acaccgttta
  -------->
601 ccttgcttat agcactaaaa ttaattattg tgtcatagca taataaataa tactggtgct

661 tcatgagaga attagttgta aaagacaatg ccttaattaa tgcaagctat aacttagatt
          repB
721 tagtagaaca acgtttaatt ttattggcca ttgttgaggc aagagaaagc gggaaaggta

Iteron 1 (520..541): atatgacaccatttaccttgca (22 nt)
Iteron 2 (542..563): atatgacaccgtttaccttgca
Iteron 3 (564..585): atatgacaccgtttaccttgca
Iteron 4 (586..607): atatgacaccgtttaccttgct
```

pMMCU3 (NC_019199.1; 8964 bp) (*reverse complement*)

```
cattcatcca ggttaaaaat ttcaacccaa aactttaatt atgaaaagct tcacagaaag 7741
   hypothetical ORF
cgttcaaatg cgatttaaga gcctttactt aaaaaacata gatctcatag tgaaaaacag 7681
aaaacagccc aaaaaacgca aatgagaacg aaataaagag agattttaac tttagataga 7621
tgcatagagc gagtgtctac gagcgaacta tcaaaatttg cgcctagacc ctctgaaaaa 7561
cattttttttg tcctctttag cctaagaaag cgtaagtttc atacagaaat ttgctcctgg 7501
atcgagcgta gcgagtaaaa aagttttatg agcgaagcga attccgagtt gcttttgctt 7441
tttcttaaag tcacgcaagt attaaccaaa aaattgcccc aacgaactga gcgaaagcga 7381
agttcaatag agtttgagcg aagcgaaaac caagggcaat ttttcattcc ctgggctttt 7321
aattattttt aatcttttaa atgcttttag acatgctgaa agccttaatt tacaacactt 7261
        ------------------------->------------------------->---------
tcaaaggcta tatgacacca tttaccttgc aatatgacac cgtttacctt gcaatatgac 7201
---------------->------------------------->
accgtttacc ttgcaatatg acaccgttta ccttgcttat agcactaaaa ttaattattg 7141
tgtcatagca taataaataa tactggtgct tcatgagaga attagttgta aaagacaatg 7081
                                             repB
ccttaattaa tgcaagctat aacttagatt tagtagaaca acgtttaatt ttattggcca 7021

Iteron 1 (7251..7230): atatgacaccatttaccttgca (22 nt)
Iteron 2 (7229..7208): atatgacaccgtttaccttgca
Iteron 3 (7207..7186): atatgacaccgtttaccttgca
Iteron 4 (7185..7164): atatgacaccgtttaccttgct
```

pAbATCC329 (NC_025168.1; 8842 bp) (*reverse complement*)

```
tccattcatc caggttaaaa atttcaaccc aaaactttaa ttatgaaaag cttcacagaa 7621
        hypothetical ORF
agcgttcaaa tgcgatttaa gagcctttac tttaaagaga gattttaact ttagatagat 7561
gcatagagcg agtgtctacg agcgaactat caaaatttgc gcctagaccc tctgaaaaac 7501
atttttttgt cctctttagc ctaagaaagc gtaagtttca tacagaaatt tgctcctgga 7441
tcgagcgtag cgagtaaaaa agttttatga gcgaagcgaa ttccgagttg cttttgcttt 7381
ttcttaaagt cacgcaagta ttaaccaaaa aattgcccca acgaactgag cgaaagcgaa 7321
gttcaataga gtttgagcga agcgaaaacc aagggcaatt tttcattccc tgggctttta 7261
attattttta atcttttaaa tgcttttaga catgctgaaa gccttaattt acaacacttt 7201
          -------------------------> ------------------------>----------
caaaggctat atgacaccat ttaccttgca atatgacacc gtttaccttg caatatgaca 7141
--------------->------------------------>
ccgtttacct tgcaatatga caccgtttac cttgcttata gcactaaaat taattattgt 7081
gtcatagcat aataaataat actggtgctt catgagagaa ttagttgtaa aagacaatgc 7021
                                         repB
cttaattaat gcaagctata acttagattt agtagaacaa cgtttaattt tattggccat 6961

Iteron 1 (7192..7171): atatgacaccatttaccttgca (22 nt)
Iteron 2 (7170..7149): atatgacaccgtttaccttgca
Iteron 3 (7148..7127): atatgacaccgtttaccttgca
Iteron 4 (7126..7105): atatgacaccgtttaccttgct
```

GR20 group

pABVA01 (NC_012813.1; 8963 bp)

```
8881 aaaaaagtat ttatttcctt gtttagaaaa acgtaaaaaa tcaatttttt gtccattcat
8941 ccaggttaaa aatttcaacc caa
        hyp. ORF
   1 aactttaatt atgaaaagct tcacagaaag cgttcaaatg cgatttaaga gcctttactt
  61 aaaaaacata gatctcatag tgaaaaacag aaaacagccc aaaaaacgca aatgagaacg
 121 aaataaagag agattttaac tttagataga tgcatagagc gagtgtctac gagcgaacta
 181 tcaaaatttg cgcctagacc ctctgaaaaa cattttttttg tcctctttag cctaagaaag
 241 cgtaagtttc atacagaaat ttgctcctgg atcgagcgta gcgagtaaaa aagttttatg
 301 agcgaagcga attccgagtt gcttttgctt tttcttaaag tcacgcaagt attaaccaaa
 361 aaattgcccc aacgaactga gcgaaagcga agttcaatag agtttgagcg aagcgaaaac
 421 caagggcaat ttttcattcc ctgggctttt aattattttt aatcttttaa atgcttttag
                                                     ------------------------
 481 acatgctgaa agccttaatt tacaacactt tcaaaggcta tatgacacca tttaccttgc
   ->------------------------>------------------------>-------------------
 541 aatatgacac cgtttacctt gcaatatgac accgtttacc ttgcaatatg acaccgttta
   -------->
 601 ccttgcttat agcactaaaa ttaattattg tgtcatagca taataaataa tactggtgct
 661 tcatgagaga attagttgta aaagacaatg ccttaattaa tgcaagctat aacttagatt
            repB
 721 tagtagaaca acgtttaatt ttattggcca ttgttgaggc aagagaaagc gggaaaggta
```

```
Iteron 1 (520..541): atatgacaccatttaccttgca (22 nt)
Iteron 2 (542..563): atatgacaccgtttaccttgca
Iteron 3 (564..585): atatgacaccgtttaccttgca
Iteron 4 (586..607): atatgacaccgtttaccttgct
```

pABUH6b-10 (AYFI01000019.1; 10030 bp)

```
2221 tgtgtggatg agattaaagc agaacaacag atgaaaatca gacaagaaca acaactcaaa
2281 gcacagcaac atgcgcctaa aatgaaatct cgtggcatga gtcgttaaac agagcgagtg
                                   mobA/mobL      ***
2341 tctacgagcg aactgagaaa atttgcccat ccctttccct gattacaagc tccctatcca
2401 tcaatcacct gcataccaga gcatcccgca tgggtgcgaa ctttccaagt ttgattccta
2461 aatcgagcgt agcgagaaaa aaagctcatg agcgaagcga attccgagtt gcttttgatc
2521 ttgcttttgc tttttctcaa attcacgaaa atatgaacca aaaattgccc cgacgaatcg
2581 agcgaaagcg agattcaata gagtttgagc gaacgaagtg aagcgaaaac caagggcaat
2641 ttttcactcc ttgggctttt aattatttta aagtttttaa atgcttttag atatgctgaa
2701 agcctttgtt cacaatgctt ttaaggttta tatgacaccg tttaccttgc aatatgacac
2761 cgtttacctt gcaatatgac accgtttacc ttgcaatatg acaccgttta ccttgcttat
2821 agcactaaaa ttaattattg tgtcatagca taataattaa tactggtgct tcatgagaga
                                                          repB
2881 attagtggta aaagacaatg ccttaatcaa tgcaagctat aacttagatt tagtagaaca
```

```
Iteron 1 (2730..2751): atatgacaccgtttaccttgca (22 nt)
Iteron 2 (2752..2773): atatgacaccgtttaccttgca
Iteron 3 (2774..2795): atatgacaccgtttaccttgca
Iteron 4 (2796..2817): atatgacaccgtttaccttgct
```

pNaval18-7.0 (NZ_AFDA02000011.1; 7032 bp)

```
3961 aaatgaaaat aacaacgatt accgaccttg gtgaatgctg tttaaacgca aaaaaatagg
                          mobA/mobL  ***
4021 gtttttagcg atgttttgct aaaccacagt agattttata gttgagttaa gaaaaaactc
4081 ttaaatcgca aatgagagca tttagcgagt gtctacgagc gaactacagg gatctaaatt
4141 ttttcacctg tttttccctg attacaggct cccctgtgta tcaagaactc agaatcagag
4201 catcccgaat gggtgcgaac tgattacggt ggctttatgt cgagcgaagc gagtgaaaaa
4261 gttctatgag cgaagcgaat tcctagttgc ttttcttttt taaaaagtca cgaaatttta
4321 agccaaaaat tgcccctacg aactgagcga aagcgaagtt caatagagtt tgagcgtagc
4381 gaaaacatag ggcaattttt ccttgcctgg gcttttaatt attttaaagt ttttaaatgc
4441 ttttaggtaa gctgaaagcc ttgtaaataa aggcttctag acttcatatg acaccgttta
4501 ccttgcaata tgacaccgtt taccttgcaa tatgacaccg tttaccttgc aatatgacac
4561 cgtttacctt gcttatagca ctaaaattaa ttaaggtgtc atagcataat aaataatact
4621 ggtgcttcat gagagagtta gttgtaaaag acaatgcctt aattaatgca agttataact
                repB
```

```
Iteron 1 (4486..4507): atatgacaccgtttaccttgca (22 nt)
Iteron 2 (4508..4529): atatgacaccgtttaccttgca
Iteron 3 (4530..4551): atatgacaccgtttaccttgca
Iteron 4 (4552..4573): atatgacaccgtttaccttgct
```

GR20 group

Multiple sequence alignment of GR20 iterons:

```
                       Iteron 1 (520..541)   : atatgacaccatttaccttgca
pCR17-C                Iteron 2 (542..563)   : atatgacaccgtttaccttgca
                       Iteron 3 (564..585)   : atatgacaccgtttaccttgca
                       Iteron 4 (586..607)   : atatgacaccgtttaccttgct

                       Iteron 1 (7251..7230): atatgacaccatttaccttgca
pMMCU3                 Iteron 2 (7229..7208): atatgacaccgtttaccttgca
                       Iteron 3 (7207..7186): atatgacaccgtttaccttgca
                       Iteron 4 (7185..7164): atatgacaccgtttaccttgct

                       Iteron 1 (7192..7171): atatgacaccatttaccttgca
pABTCC329              Iteron 2 (7170..7149): atatgacaccgtttaccttgca
                       Iteron 3 (7148..7127): atatgacaccgtttaccttgca
                       Iteron 4 (7126..7105): atatgacaccgtttaccttgct

                       Iteron 1 (520..541)   : atatgacaccatttaccttgca
pABVA01                Iteron 2 (542..563)   : atatgacaccgtttaccttgca
                       Iteron 3 (564..585)   : atatgacaccgtttaccttgca
                       Iteron 4 (586..607)   : atatgacaccgtttaccttgct

                       Iteron 1 (2730..2751): atatgacaccgtttaccttgca
pABUH6b-10             Iteron 2 (2752..2773): atatgacaccgtttaccttgca
                       Iteron 3 (2774..2795): atatgacaccgtttaccttgca
                       Iteron 4 (2796..2817): atatgacaccgtttaccttgct

                       Iteron 1 (4486..4507): atatgacaccgtttaccttgca
pNaval18-7.0           Iteron 2 (4508..4529): atatgacaccgtttaccttgca
                       Iteron 3 (4530..4551): atatgacaccgtttaccttgca
                       Iteron 4 (4552..4573): atatgacaccgtttaccttgct
```

Iteron sequences of representative plasmids from all eight GR groups of Rep-3 family plasmids in relation to the RepB phylogenetic tree.

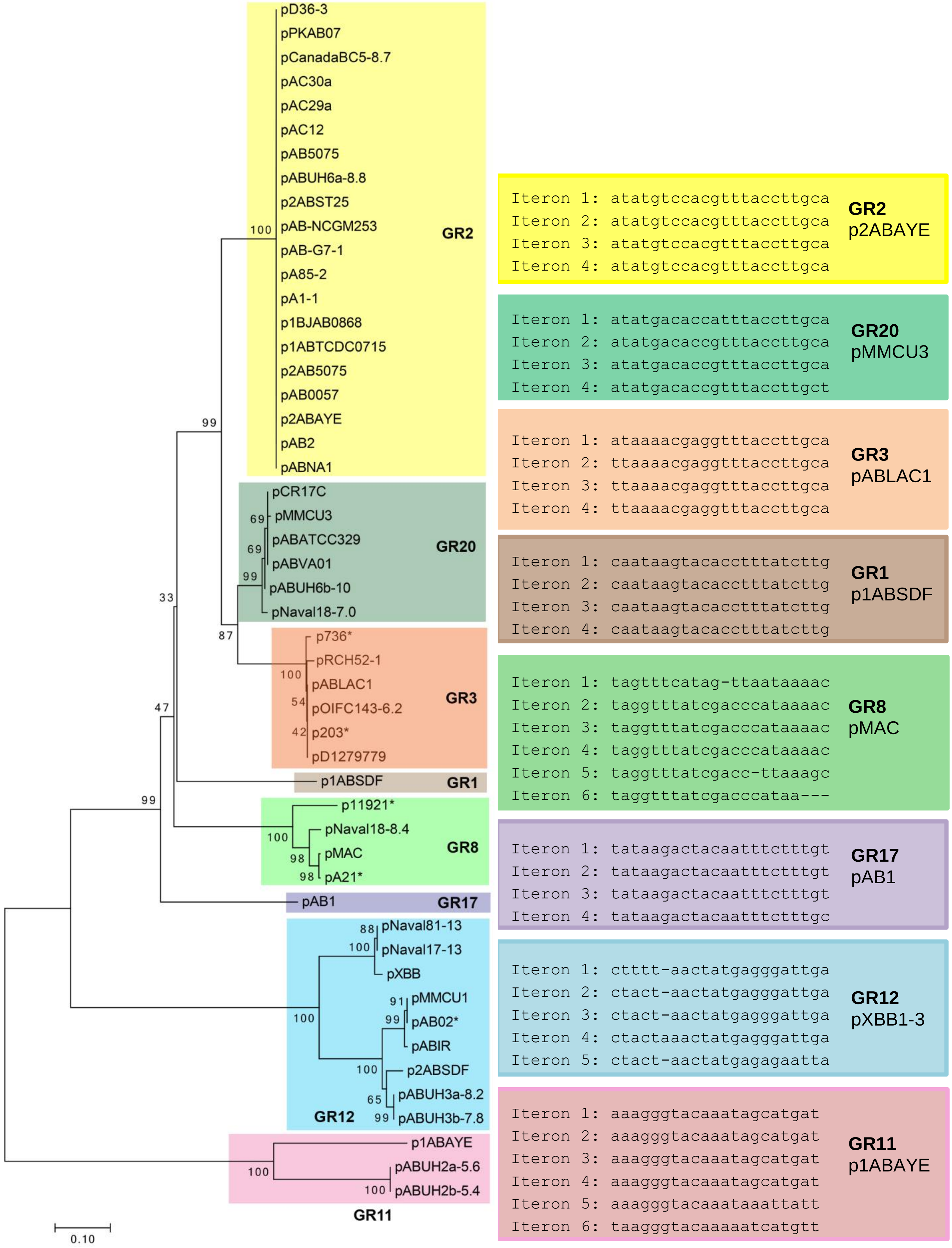

Supplement: Supplementary file 4 [file Data_Sheet_1.PDF]
